# A Retrospective Approach to Testing the DNA Barcoding Method

**DOI:** 10.1371/journal.pone.0077882

**Published:** 2013-11-11

**Authors:** David G. Chapple, Peter A. Ritchie

**Affiliations:** 1 School of Biological Sciences, Monash University, Clayton, Victoria, Australia; 2 Allan Wilson Centre for Molecular Ecology and Evolution, School of Biological Sciences, Victoria University of Wellington, Wellington, New Zealand; University of Vermont, United States of America

## Abstract

A decade ago, DNA barcoding was proposed as a standardised method for identifying existing species and speeding the discovery of new species. Yet, despite its numerous successes across a range of taxa, its frequent failures have brought into question its accuracy as a short-cut taxonomic method. We use a retrospective approach, applying the method to the classification of New Zealand skinks as it stood in 1977 (primarily based upon morphological characters), and compare it to the current taxonomy reached using both morphological and molecular approaches. For the 1977 dataset, DNA barcoding had moderate-high success in identifying specimens (78-98%), and correctly flagging specimens that have since been confirmed as distinct taxa (77-100%). But most matching methods failed to detect the species complexes that were present in 1977. For the current dataset, there was moderate-high success in identifying specimens (53-99%). For both datasets, the capacity to discover new species was dependent on the methodological approach used. Species delimitation in New Zealand skinks was hindered by the absence of either a local or global barcoding gap, a result of recent speciation events and hybridisation. Whilst DNA barcoding is potentially useful for specimen identification and species discovery in New Zealand skinks, its error rate could hinder the progress of documenting biodiversity in this group. We suggest that integrated taxonomic approaches are more effective at discovering and describing biodiversity.

## Introduction

The ability to accurately identify and describe species underpins all biological research, yet the traditional morphological-based taxonomic approaches have only managed to describe 1.2-1.5 million species over the past 250 years [1,2], a mere 10% of the Earth’s predicted eukaryotic diversity [2]. It is estimated that persisting with such time-consuming and cumbersome approaches would not result in a comprehensive inventory of the world’s biodiversity for at least ~1000 years [3,4], and perhaps much longer given the sharp decline in the number of specialist taxonomists and funding for taxonomic research [5,6]. The DNA barcoding approach was introduced in 2003 by Paul Hebert and colleagues [7,8] as a way to overcome the existing taxonomic ‘impediment’ or ‘bottleneck’ [7,9]. It promised to revolutionise the identification of existing species and speed the discovery of new species, using a standardised molecular marker (a 650bp segment of the cytochrome c oxidase I [COI] mitochondrial DNA gene) and analysis method.

In contrast to the limited number of discrete morphological characters available for identifying and discriminating species, the four alternate nucleotides (A, T, C, G) and 650 nucleotide positions in the COI gene provide an almost infinite number of potential combinations [7]. Unlike many morphological characters which are relevant only to specific taxonomic groups, sequence data is comparable across the entire animal kingdom [4], prompting the analogy to the Universal Product Codes (UPC) that uniquely identify commercial products and the suggestion that species could be identified based on their COI ‘barcode’ [7]. A significant advantage of the approach is that it works in situations that would confound many morphological approaches: specimen fragments [10-12], species with multiple life stages [13], and sexual dimorphism or conserved, variable or plastic morphology [14-16]. Importantly, advances in high-throughput sequencing technology continue to increase the speed, and decrease the cost, of generating COI reference libraries for the world’s fauna [17,18]. 

The ability to delimit species is an essential component of both species identification (hereafter referred to as specimen identification; [19]) and species discovery. A critical assumption of the DNA barcoding approach is that the level of intraspecific genetic variation is less than that evident among species [7,8,20]. This distinction between intra- and inter-specific divergences, termed the ‘barcoding gap’ [21], enables unknown sequences to be assigned to an existing species or flagged as a suspected new species. The threshold above which a query sequence is considered as distinct from a reference sequence has variously been suggested as 2-3% [7,8], 10x the mean intraspecific divergence [20], or calculated independently for each empirical dataset (e.g. Automatic Barcode Gap Discovery [ABGD], [22]). Research over the past decade has demonstrated that the accuracy of species delimitation is influenced by the quality and completeness of the reference database, the geographic extent of sampling, the intensity of intraspecific sampling, and the timing of divergence among closely-related species [23-27].

The traditional DNA barcoding approach relies upon a single mtDNA gene region and experiences inherent difficulties in instances of introgression, incomplete lineage sorting, pseudogenes, gene duplication, horizontal gene transfer, and mtDNA selection [28-32]. Despite these acknowledged limitations, continued development of the DNA barcoding method (e.g. improved analytical and statistical approaches, the use of other mtDNA and nuclear gene regions) have enabled it to gain widespread acceptance with several international collaborations and consortiums dedicated to barcoding all animal life (BOLD, CBOL, iBOL). Yet, the numerous purported successes of the method [20,26,33-41] have been tempered by its frequent failure across a broad range of animal groups [21,25,42-48]. Few studies have reported complete (i.e. 100%) success [35], and often the basis on which a particular study is designated as a success or failure is subjective. 

Aside from instances involving inherent methodological issues (e.g. introgression, incomplete lineage sorting), on some occasions ‘failures’ are reported as successes, with researchers: i) questioning the quality or reliability of the existing reference datasets [4], or ii) citing instances of intra- and inter-specific overlap as evidence (or proof) for the presence of species complexes or distinct taxa that have been overlooked previously [20,26,33,49]. This has led critics to label DNA barcoding as a method that is set-up so that it ‘cannot fail’ [50]. Only a subset of studies subsequently use integrated taxonomic approaches to assess the validity of flagged species complexes and suspected new taxa, making it difficult to determine whether they represent problems with the existing taxonomy [16,26,51] or the DNA barcoding approach itself [48,52]. Distinguishing between these alternate possibilities is often difficult as the ‘true’ taxonomy is generally unknown, and represents the actual focus of the DNA barcoding study. Here we outline a case study where we use a retrospective approach that enables us to assess the potential value of the DNA barcoding method as a short-cut approach to specimen identification and species discovery in New Zealand lizards.

We apply the DNA barcoding approach to the New Zealand skink fauna (Scincidae) as it stood prior to the implementation modern molecular techniques [53], and compare it to the current taxonomy that has been reached following 25 years of intensive, integrated taxonomic study [54-76]. In 1977, Hardy [53] completed a comprehensive, primarily morphologically-based revision of New Zealand skinks, recognising 23 distinct species or taxa (Table S1). The conserved morphology of this radiation has led to a turbulent taxonomic history, but Hardy’s [53] revision provides a convenient baseline of the taxonomic resolution possible from morphological characters. Studies combining morphological and molecular (allozymes, mtDNA, nuclear DNA) approaches have resulted in the splitting of species complexes and discovery of new taxa, leading to the current recognition of 55 species, several of which remain to be formally described (Table S1; [66,75]). 

This approach enables us to: i) determine whether species complexes in the 1977 dataset are correctly identified and flagged, ii) compare the outcome of the DNA barcoding method on the current dataset with that reached through an integrated taxonomic approach, and iii) assess whether purported new taxa discovered since 1977 can be correctly assigned as new or existing taxa through DNA barcoding. This will be achieved using 296 New Zealand skink COI sequences representing all 23 taxa recognised in 1977 (1-82 sequences per species) and 48 of the 55 currently recognised species (1-27 sequences per species) (Table S2). Our study represents one of the few DNA barcoding studies conducted on reptiles ([18], but see 77).

## Materials and Methods

### Sampling and COI sequencing

Samples were obtained, with permission, from the National Frozen Tissue Collection (NFTC, Victoria University of Wellington, New Zealand; associated voucher specimens are housed at Te Papa) and ethanol-preserved specimens housed at Te Papa Tongarewa (National Museum of New Zealand, Wellington) (Table S2). These institutions donated the tissue samples for use in this study. The only extant species not included were four recently described species (*O. burganae*, *O. judgei*, *O. repens*, *O. toka*; [67,74]), a recently recognised undescribed species (*O*. aff. *polycroma* ‘Clade 2’; [70]), and two presumed distinct taxa (each known only from a single specimen: *O*. aff. *inconspicuum* ‘Okuru’ [67]; *O*. ‘Whirinaki’ [75]). For the phylogenetic analyses, outgroup samples from New Caledonia and Australia were included, based on broader phylogenetic studies of Eugongylus Group skinks in the region [66] (Table S2).

Total genomic DNA was extracted from liver, muscle or tail samples using a modified phenol-chloroform extraction protocol [78]. Skink specific primers were developed to amplify and sequence a ~710bp fragment of the COI mtDNA gene (Table S3). PCR and sequencing were conducted as outlined in Greaves et al. [61]. PCR products were purified using ExoSAP-IT (USB Corporation, Cleveland, Ohio USA). The purified product was sequenced directly using a BigDye Terminator v3.1 Cycle Sequencing Kit (Applied Biosystems) and then analysed on an ABI 3730XL capillary sequencer. Sequence data were edited and aligned using Geneious v5.4 [79]. We translated all sequences to confirm that none contained premature stop codons. The sequence data were submitted to GenBank under accession number KC349552-KC349853 (Table S2).

### Barcoding gap analyses

Intra- and inter-specific genetic distances were calculated in MEGA5 [80] using the Kimura 2-Parameter model (K2P). Species Identifier v1.7.8 (http://taxondna.sourceforge.net/; [43]) was used to calculate the level of overlap (total and 90%) between the intra-specific and inter-specific genetic distances. The program ABGD (http://wwwabi.snv.jussieu.fr/public/abgd/abgdweb.html; [22]) was used to determine the genetic distance threshold for species delimitation, following the methodology of Jörger et al [51] and Puckridge et al. [41].

As some overlap between intra- and inter-specific divergences has become the expectation, rather than a rare exception [21,46], its presence does not necessarily preclude specimen identification [19]. This is because specimen identification relies upon the presence of a ‘local’ barcoding gap (i.e. a query sequence being closer to a conspecific sequence than a different species), rather than the ‘global’ barcoding gap (i.e. a distance threshold set for all species) that is required for species discovery [19,46]. Plots of maximum intra-specific distance against minimum inter-specific distance (i.e. nearest neighbour; [81]) are used to investigate the presence of a local barcoding gap, with points below the 1:1 slope representing instances where it is absent [19,26,40,49]. We used Analysis of Variance (ANOVA) to compare the level of intraspecific divergence present in the 1977 and current taxonomy datasets.

### Specimen identification

So as to not confound specimen identification and species discovery [19], we consider each separately. We employed three different approaches to specimen identification: Neighbour-Joining (NJ)-based, distance-based, and matching methods.

#### NJ-based method

This involved the modified version of the NJ method of Hebert et al. [7], originally developed by Meier et al. [43] (“tree-based identification, revised criteria”). NJ trees were generated in MEGA using K2P genetic distances and 1000 bootstraps. An exemplar sequence was designated for each species [35,46], representing (where possible) the sample that was geographically closest to the type locality for the species (Table S2). The criteria outlined in Table S4 were used to determine whether specimen identification was successful, ambiguous, or a failure (i.e. mis-identification). For the 1977 dataset, we compared the specimens listed as ambiguous to the current taxonomy to determine whether they were correctly flagged as representing new species. 

#### Distance-based method

A dataset was generated containing only the exemplar sequence for each species. Using Species Identifier, we calculated the genetic distance to the nearest exemplar sequence for each query specimen. Identification success or failure was assessed against a series of distance thresholds (2, 4, 6, 8, 10%). For the 1977 dataset, we determined whether the specimen queries that exceeded the respective distance threshold were correctly flagged as new/distinct species relative to the current taxonomy. 

#### Matching methods

Species Identifier was used to determine the success of three matching approaches (“Best Match”, “Best Close Match”, “All Species Barcode”) originally developed by Meier et al. [43]. The criteria used to assess whether specimen identification was successful, ambiguous, or a failure are outlined in Table S4. 

### Species discovery

Thirteen suspected new taxa (morphologically distinct forms, or discoveries from remote regions of the South Island) have been discovered in New Zealand since 1977 (Table S5). Integrated taxonomic approaches have confirmed some as new species, while others simply represent morphologically distinct forms of existing species [66,75] (Table S5). We used the 1977 dataset, and a modified version of the current taxonomy dataset (containing only taxa/populations known in 1977), to assess whether the NJ-based and distance-based approaches would have correctly identified these suspected new taxa as new or existing species.

## Results

### Barcoding gap analyses

**Figure 1:**
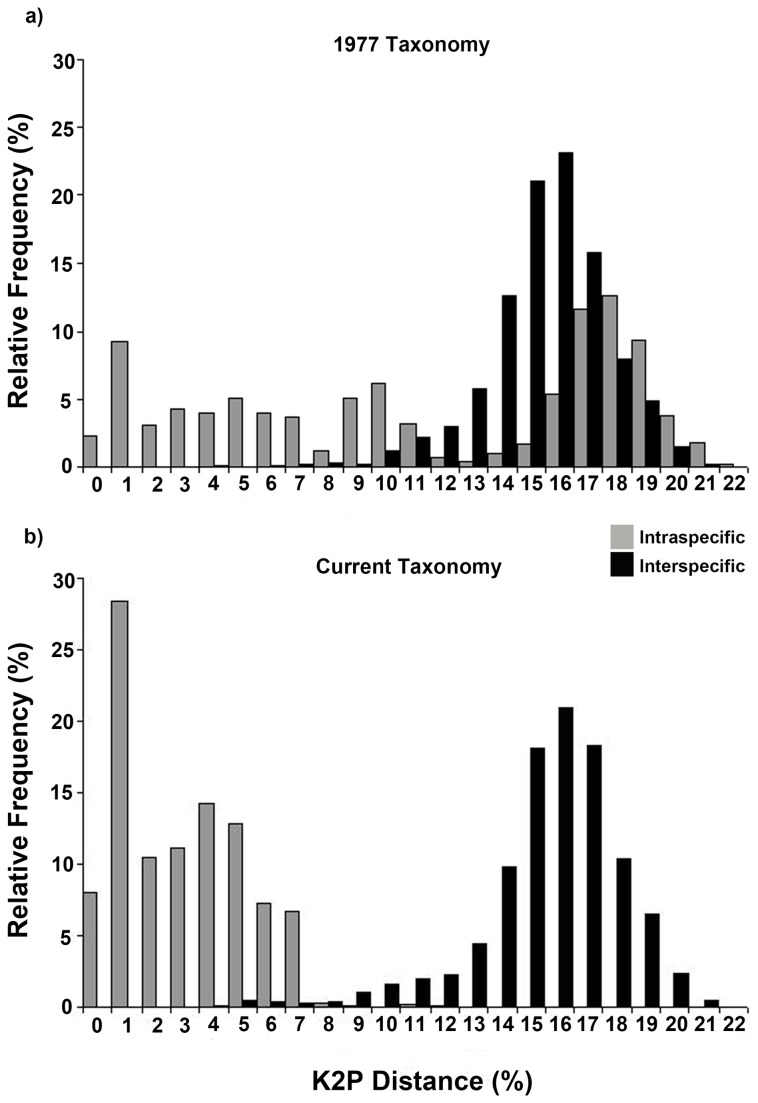
The barcoding gap, the overlap of intra- and inter-specific K2P genetic distances. Based on the (*A*) 1977 taxonomy, and (*B*) current taxonomy of New Zealand skinks.

Both the mean (1977: 3.3 ± 0.75, Current: 1.9 ± 0.29; ANOVA: *F*_1,61_ = 4.57, *P* = 0.037) and maximum (1977: 5.6 ± 1.25, Current: 3.0 ± 0.45; ANOVA: *F*_1,61_ = 5.51, *P* = 0.022) intraspecific genetic distances were higher under the 1977 taxonomy compared to the current taxonomy (Table S5, Table S6). This resulted in near complete overlap between intra- and inter-specific genetic distances for the 1977 dataset, and the absence of a barcoding gap (Figure 1, Table 1). Although some overlap was still evident under the current taxonomy (4-10% of observations), there was a clearer distinction between the intra- and inter-specific genetic distances (Figure 1, Table 1).

**Table 1:** Overlap in the intra- and inter-specific genetic distances in New Zealand skinks.

Dataset	No. samples	Total Overlap		90% Overlap	
		Overlap (range)	% Observations	Overlap (range)	% Observations
1977 taxonomy	256	21.28% (0-21.28%)	99.9%	8.49% (10.68-19.17%)	88.1%
Current taxonomy	296	11.03% (0-11.03%)	10.4%	4.05% (6.26-10.31%)	4.1%

Based on the 1977 taxonomy and current taxonomy. The 90% overlap excludes the largest 5% of the intra-specific distances and the lowest 5% of the inter-specific distances.

**Figure 2:**
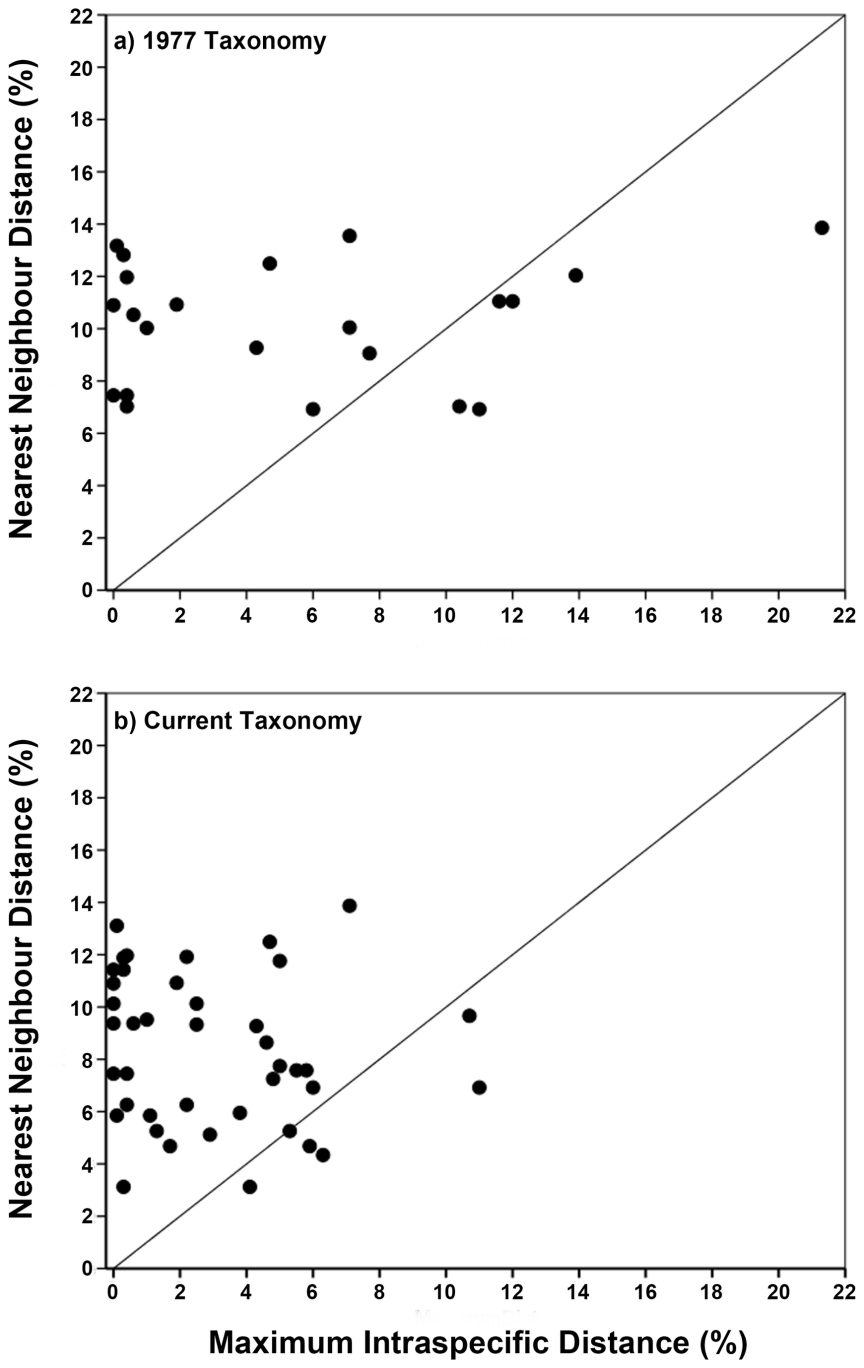
Maximum intra-specific K2P genetic distance in relation to the nearest neighbour distance. Based on the (*A*) 1977 taxonomy for New Zealand skinks, and (*B*) current taxonomy. Points that fall above the 1:1 line indicate the presence of a local barcode gap, whereas this local barcode gap is absent for the points below the line.

For the 1977 taxonomy, a local barcoding gap was not present for six species (27%; Figure 2). Five of these instances related to species complexes that have been split since 1977 (*aeneum*, *lineoocellatum*-*chloronoton*, *oliveri*, *nigriplantare maccanni*; Table S1), and one to hybridisation among *otagense* and *waimatense* [68]. However, even though these complexes have been revised in the current taxonomy, a local barcoding gap was absent for six species (5 [12%] below the line, 1 [2%] on the line; total 14%; Figure 2). These instances involved recent speciation (*infrapunctatum*, *lineoocellatum*-*chloronoton*, *smithi-Microlepis*; [61,62,69]) and hybridisation among *otagense* and *waimatense* [68]. 

### Specimen identification

We implemented several approaches to determining the distance threshold for species delimitation. The 10x mean intraspecific divergence approach yielded unreasonably high thresholds for both the 1977 (33.1%) and current datasets (18.7%). Although the results of the ABGD approach were somewhat inconclusive, the most consistent threshold range was 2.3-3.8%. Thus, we used a broad range of distance thresholds (2, 4, 6, 8, 10%) to assess the accuracy of specimen identification using NJ-based, distance-based (distance to species exemplar sequence) and matching methods (best match, best close match, all species barcode).

#### NJ-based

The NJ approach correctly identified 56% of specimens as existing species, with a further 42% flagged as distinct species (of these, 97% have subsequently been confirmed as new species) ([Table pone-0077882-t002], Figure S1). This resulted in an overall success rate of 97%, similar to that found based on the current taxonomy (96%; Table 2, Figure S2). The instances of failure related to hybridisation (*otagense-waimatense*) and recent species radiations (Figure S1, Figure S2). 

**Table 2 pone-0077882-t002:** Success rate for the NJ-based (Neighbour-Joining) approach to specimen identification and species discovery.

	1977 Taxonomy	Current Taxonomy
**Specimen identification**		
Success	56% (130)	96% (237)
Ambiguous	42% (98)	2% (7)
*Correctly flagged?*	*41% (95*)	*NA*
Misidentified	2% (5)	1% (4)
**Species Discovery- new taxa since 1977**		
Success	100% (40)	100% (40)
Correctly listed as existing species	4	29
Correctly flagged as new species	11	11
Correctly flagged, but part of known complex	25	NA

Based on the 1977 taxonomy and current taxonomy.

#### Distance to species exemplar

The specimen identification ‘success’ (= correct exemplar, within threshold + correctly flagged as new species) for the 1977 dataset ranged between 78-89%, depending on the threshold used (Table 3). The lower thresholds were more successful at flagging new species, but conversely had higher error rates for incorrectly flagging specimens are distinct (Table 3). Identification success for the current dataset ranged between 53-96%, with the rate of incorrect flagging decreasing with the threshold employed (Table 3).

**Table 3:** Specimen identification success in New Zealand skinks.

Method	1977 Taxonomy					Current Taxonomy				
	2%	4%	6%	8%	10%	2%	4%	6%	8%	10%
**SPECIMEN IDENTIFICATION**										
**Distance to Exemplar**										
Correct exemplar, within threshold	39% (91)	47% (111)	54% (126)	58% (136)	59% (137)	53% (131)	80% (198)	92% (229)	95% (237)	96% (238)
Correctly flagged as new species	39% (92)	39% (90)	35% (82)	30% (69)	26% (62)	NA	NA	NA	NA	NA
Incorrectly flagged as new species	21% (48)	12% (28)	6% (13)	1% (3)	1% (2)	46% (115)	16% (40)	4% (9)	1% (1)	0% (0)
ID as wrong species/Not flagged as new	1% (2)	2% (4)	5% (12)	11% (25)	14% (32)	1% (2)	4% (10)	4% (10)	4% (10)	4% (10)
**Best Match**										
Success, within threshold	86% (219)	95% (241)	96% (246)	98% (249)	98% (250)	88% (256)	96% (279)	98% (283)	98% (284)	99% (285)
Success, outside threshold	12% (31)	3% (9)	2% (4)	>1% (1)	0% (0)	10% (29)	2% (6)	1% (2)	<1% (1)	0% (0)
Ambiguous	1% (3)	1% (3)	1% (3)	1% (3)	1% (3)	1% (3)	1% (3)	1% (3)	1% (3)	1% (3)
Misidentification	1% (2)	1% (2)	1% (2)	1% (2)	1% (2)	<1% (1)	<1% (1)	<1% (1)	<1% (1)	<1% (1)
**Best Close Match**										
Success	86% (219)	95% (241)	96% (246)	98% (249)	98% (250)	88% (256)	96% (279)	98% (283)	98% (284)	99% (285)
Ambiguous	1% (3)	1% (3)	1% (3)	1% (3)	1% (3)	1% (3)	1% (3)	1% (3)	1% (3)	1% (3)
Misidentification	<1% (1)	<1% (1)	<1% (1)	<1% (1)	<1% (1)	<1% (1)	<1% (1)	<1% (1)	<1% (1)	<1% (1)
No Match	13% (32)	4% (10)	2% (5)	1% (2)	<1% (1)	10% (29)	2% (6)	1% (2)	<1% (1)	0% (0)
**All Species Barcode**										
Success	26% (67)	29% (75)	30% (76)	30% (76)	30% (76)	63% (183)	68% (197)	69% (201)	70% (202)	70% (202)
Ambiguous	61% (156)	67% (170)	68% (174)	69% (177)	70% (178)	27% (77)	30% (86)	30% (86)	30% (86)	30% (87)
Misidentification	0% (0)	0% (0)	0% (0)	0% (0)	0% (0)	0% (0)	0% (0)	0% (0)	0% (0)	0% (0)
No Close Match	13% (32)	4% (10)	2% (5)	1% (2)	<1% (1)	10% (29)	2% (6)	1% (2)	<1% (1)	0% (0)
**SPECIES DISCOVERY**										
**Distance to Exemplar**										
Success	**95%**	**95%**	**82%**	**82%**	**77%**	**35%**	**80%**	**87%**	**87%**	**77%**
Correctly grouped with existing species	2	2	2	2	2	3	21	29	29	29
Correctly flagged as a new species	11	11	6	6	4	11	11	6	6	2
Correctly flagged, but part of a known complex	25	25	25	25	25	0	0	0	0	0
Failure	**5%**	**5%**	**18%**	**18%**	**23%**	**65%**	**20%**	**13%**	**13%**	**23%**
Incorrectly flagged as a new species	2	2	2	2	2	26	8	0	0	0
Incorrectly lumped with an existing species	0	0	5	5	7	0	0	5	5	9

Using the distance-based (Distance to Nearest Exemplar) and matching methods (Best Match, Best Close Match, All Species Barcode) for New Zealand skinks based on the 1977 taxonomy and current taxonomy. Identification success was assessed using a range of K2P distance thresholds (2, 4, 6, 8, 10%). The effectiveness of the distance-based approach for species discovery was also investigated for the new taxa found since 1977.

#### Matching methods

Despite the taxonomic issues evident in the 1977 dataset, high levels of success (86-98%) were reported with the Best Match and Best Close Match approaches (Table 3). The rate of success was similar in the current dataset (88-99%), with the instances of failure generally involving hybridisation (*otagense-waimatense*) or recent radiations (Table 3).

In contrast, the All Species Barcode approach was much more effective at identifying the taxonomic issues present in 1977, with a low success rate (26-30%) reported across all distance thresholds (Table 3). Yet, only moderate success (63-70%) was evident for the current taxonomy, due to a high number of ambiguous sequences stemming from the absence of a local or global barcoding gap (Table 3).

### Species discovery

To examine the effectiveness of the DNA barcoding approach for species discovery, modified versions of both datasets were used that contained only taxa that were known in 1977. The NJ method correctly assigned all (100% success) suspected new taxa discovered since 1977 as either part of an existing species or correctly flagged as a new species (Table 2). The Distance to Species Exemplar approach was less accurate, with the success depending on the dataset used and the distance threshold employed (1977: 77-95%, Current: 35-87%; Table 3).

## Discussion

### Barcoding gap

We failed to find evidence for either a local or global barcoding gap for New Zealand skinks. The presence of a barcoding gap is essential for accurate species delimitation, and underlies both specimen identification (local gap) and species discovery (global gap) [19,46]. Overlap between intra- and inter-specific genetic distances is often attributed to issues with the existing taxonomy or quality of the reference dataset [20,49]; however, our retrospective approach enables us to exclude these as explanations for the absence of a barcoding gap in this group. 

For the 1977 dataset, the near complete overlap of intra- and inter-specific genetic variation was due to the widespread presence of unrecognised species complexes (Table S1). Yet, while these complexes were resolved in the current taxonomy, due to hybridisation (*otagense* and *waimatense*; [68]) and several recent speciation events (*infrapunctatum*, *lineoocellatum*-*chloronoton*, *smithi-Microlepis*; [61,62,69]) a barcoding gap was still absent. The limited potential for species delimitation in New Zealand skinks might point to potential shortcomings of the traditional (i.e. COI-only) DNA barcoding approach, rather than to issues related to the quality of existing reference taxonomies or datasets. The initial studies documenting the presence of distinct barcoding gaps in animals [7,8,20] understated the degree of intraspecific genetic divergences (due to limited sampling within species) and overstated the level of interspecific distances (due to a lack of closely-related species) [21,52,82]. Numerous empirical studies employing comprehensive sampling within taxonomic groups [21,25,26,46,49,52], including the present study, have supported theoretical predictions [23,27] that the overlap between intra- and inter-specific genetic distances is a common occurrence in recent radiations (but see 39). 

The concept of a barcoding gap is directly linked to the search for a set distance threshold at which to delimit species within DNA barcoding studies. Yet, due to factors such as population size, mutation rate and biogeographic history, there is no *a priori* reason to expect that divergence times within or among lineages will be consistent [19]. For instance, the level of intraspecific genetic variation in amphibians and reptiles differs significantly among the Northern and Southern Hemisphere’s due to divergent climatic histories over the past 5 myr’s [83,84]. For New Zealand skinks, it is known that there were several periods of speciation; the first occurring after the initial colonisation of the country ~16-19 mya, followed by more recent events in response to Pliocene tectonic uplift in the South Island and Pleistocene glacial cycles [66]. It is these more recent speciation events that have led to the absence of a local (Figure 2) or global (Figure 1, Table 1) barcoding gap in New Zealand skinks.

### Specimen identification

Our retrospective approach in New Zealand skinks highlights the importance of the quality of the reference database for specimen identification. Database quality is reliant on both i) the number of taxa sampled and the level of intra-specific sampling [24], and ii) the ‘accuracy’ of the taxonomy used [29,30,43,50]. As our study was based on detailed within-species sampling with complete (1977 dataset) or near-complete (current dataset) coverage of known taxa, we could therefore focus on the impact of taxonomic accuracy on specimen identification. 

Specimen identification success based on the 1977 taxonomy was moderate to high across the NJ-based (97%), distance-based (78-89%), and matching methods (86-98% for Best Match and Best Close Match). Importantly, the NJ- and distance-based approaches could flag specimens that were putatively distinct from known taxa. The NJ method was highly accurate (97%) in regard to which specimens it flagged as new, while the threshold used in the Distance to Exemplar approach influenced (inversely) the number (64-140) and accuracy (66-97%) of specimens flagged. However, the matching methods struggled with the taxonomic issues present in the 1977 dataset. While the All Species Barcode approach correctly detected the presence of species complexes (i.e. low success rate, 26-30%), the Best Match and Best Close Match methods reported high ‘success’ despite these taxonomic issues. This situation highlights a novel instance of the ‘closest match fallacy’ [50], whereby specimens with close ‘within threshold’ matches in the reference dataset might represent distinct taxa if unresolved species complexes are present in the group.

Our study of New Zealand skinks provides mixed support for the suggestion that the DNA barcoding approach is a valid ‘short-cut’ taxonomic method compared to either the traditional or modern, integrated approaches [9,51]. Firstly, our barcoding study was completed in only a few months compared to the two decades it has taken for the integrated modern approach (Table S1). However, as outlined previously in other studies [21,30,43,50], the barcoding approach in New Zealand skinks could not have been implemented without a strong taxonomic foundation, developed through traditional methods ([53]; Table S1). Secondly, we were unable to identify an ideal distance threshold for species delimitation. Based on the current taxonomy, high error rates (4-47%) were evident for the 2-3% and ABGD approaches, while the 10x intraspecific divergence yielded an unrealistically high threshold (18.7%). Thirdly, for the current dataset, specimen identification success ranged from moderate (53-96%, Distance to Exemplar; 63-70%, All Species Barcode) to high (88-99%, Best Match & Best Close Match; 96%, NJ tree). Although the instances of hybridisation would confound any approach relying solely on mtDNA [85], it would be easily detected by any integrated taxonomic method [28,31]. Finally, the absence of the barcoding gap (Figures 1, 2), or the selection of an inappropriate distance threshold (Table 3), could led the incorrect flagging of specimens as distinct, wasting valuable time and hindering the progress of documenting and describing the true diversity within a group [29].

### Species discovery

The capacity for the DNA barcoding approach to assist with species discovery has been one of the more contentious aspects of the method [7,8,29-31]. Yet the NJ-based method correctly assigned, as either new or existing species, all 13 taxa (represented by a total of 40 specimens) discovered in New Zealand since 1977. In contrast, the Distance to Exemplar method exhibited lower success, depending on the distance threshold (35-87%). This has important implications for the potential of this approach for species discovery, since the exemplar method relates to the specimen closest to the type location and is therefore taxonomically relevant. Given that the exemplar and matching methods also had difficulties in identifying instances of species complexes in the 1977 dataset, these approaches might have limited utility for species discovery in DNA barcoding studies. 

## Conclusions

Although DNA barcoding has not turned out to be the panacea for resolving the taxonomic impediment, there is still substantial value in the approach for biodiversity and taxonomic studies. It represents an ideal approach for conducting quick, preliminary studies of either well-characterised groups or poorly known taxonomic groups or geographic regions; with the initial barcoding study providing a framework for subsequent, and more detailed, integrated taxonomic approaches [9]. Accordingly, DNA barcoding studies are increasingly moving away from the traditional COI-only approaches and incorporating sophisticated statistical approaches to species delimitation (e.g. Bayesian Species Delineation [86,87], Generalised Mixed Yule Coalescent model [88,89], ABGD [22], Fuzzy Membership [90]), including additional mtDNA or nuclear genes [9,51], and adopting integrated taxonomic approaches [16,51,91]. In particular, character-based methods, which were not part of the original DNA barcoding approaches, have been used increasingly across a range of taxa [9,18,92-95]. In adopting these modified approaches, researchers are moving away from some elements of the initial philosophy and concepts that underpinned the DNA barcoding approach, but towards a more robust integrated method that is better equipped to address the current taxonomic impediment and speed the rate of species discovery and description.

## Supporting Information

Table S1 **Comparison of the current taxonomy for New Zealand skinks with that recognised in 1977, prior to the implementation of modern molecular techniques.** Evidence on which the current taxonomy is based: 1: allozymes, 2: mitochondrial DNA sequence data, 3: nuclear DNA sequence data, 4: morphological data, 5: proposed taxonomic change yet to be confirmed.
(PDF)

Table S2 **Locality data, museum voucher specimen information, and GenBank accession numbers for the New Zealand skink samples used in this study.** Samples with CD or FT codes were obtained from the National Frozen Tissue Collection (NFTC) housed at Victoria University of Wellington, New Zealand (the associated voucher specimens are now housed at Te Papa). Samples with RE codes were obtained from Te Papa, National Museum of New Zealand, Wellington (S codes refer to specimens from the former Ecology Division collection, now housed at Te Papa). Samples with ABTC (Australian Biological Tissue Collection) codes were obtained from the South Australian Museum. Samples with NR and EBU codes were obtained from the Australian Museum. Asterisks indicate the exemplar specimens.
(PDF)

Table S3 **Oligonucleotide primers used in this study to amplify and sequence COI in New Zealand skinks.**
(PDF)

Table S4 **Query identification criteria for the NJ-based and matching methods (modified from Meier et al. 2006).**
(PDF)

Table S5 **Number of samples and geographic localities used in the DNA barcoding study of New Zealand skinks based on the 1977 taxonomy.** (See Tables S1 and S2 for additional details.) The level (mean ± standard error [SE], and range) of intraspecific K2P genetic distances is shown for each New Zealand skink species. The sample codes (see Table S2) for the new discoveries since 1977 are indicated.
(PDF)

Table S6 **Number of samples and geographic localities used in the DNA barcoding study of New Zealand skinks (genus *Oligosoma*).** (See Table S2 for additional details.) The level (mean ± standard error [SE], and range) of intraspecific K2P genetic distances in each New Zealand skink species. The taxonomy follows the current New Zealand Threat Classification listing (Hitchmough et al. 2010).
(PDF)

Figure S1 **Neighbour-joining tree (with 1000 bootstraps) for New Zealand skinks based on the 1977 taxonomy.** Asterisks indicate the exemplar specimens for each species (See Table S2). The locality details are provided in Table S2.
(PDF)

Figure S2 **Neighbour-joining tree (with 1000 bootstraps) for New Zealand skinks based on the current taxonomy.** Asterisks indicate the exemplar specimens for each species (See Table S2). The locality details are provided in Table S2.
(PDF)
