# Supplementary material for: A Retrospective Approach to Testing the DNA Barcoding Method
Source: PLoS One. 2013 Nov 11;8(11):e77882. doi: 10.1371/journal.pone.0077882 (PMC3823873; doi:10.1371/journal.pone.0077882)
Supplement: Table S1 — Comparison of the current taxonomy for New Zealand skinks with that recognised in 1977, prior to the implementation of modern molecular techniques. Evidence on which the current taxonomy is based: 1: allozymes, 2: mitochondrial DNA sequence data, 3: nuclear DNA sequence data, 4: morphological data, 5: proposed taxonomic change yet to be confirmed. (PDF) [file pone.0077882.s001.pdf]

Table S1. Comparison of the current taxonomy for New Zealand skinks with that recognised in 1977, prior to the implementation of modern molecular techniques. Evidence on which the current taxonomy is based: 1: allozymes, 2: mitochondrial DNA sequence data, 3: nuclear DNA sequence data, 4: morphological data, 5: proposed taxonomic change yet to be confirmed.

Taxonomy- 1977	Taxonomy- Current	Evidence	Reference
Cyclodina aenea	*Oligosoma aeneum*	2-4	Chapple et al. (2008a,b, 2009)
	Oligosoma hardyi	2-4	Chapple et al. (2008a,b, 2009)
	Oligosoma levidensum	2,4	Chapple et al. (2008a,b)
Cyclodina alani	*Oligosoma alani*	2-4	Chapple et al. (2008b, 2009)
Cyclodina macgregori	*Oligosoma macgregori*	2-4	Chapple et al. (2008b, 2009)
Cyclodina oliveri	*Oligosoma oliveri*	1-4	Vos (1988), Chapple et al. (2008c, 2009)
	Oligosoma townsi	1-4	Vos (1988), Chapple et al. (2008c, 2009)
Cyclodina ornata	*Oligosoma ornatum*	2-4	Chapple et al. (2008b, 2009)
	Oligosoma aff. *ornatum* 'Poor Knights'	2-4	Chapple et al. (2008b, 2009)
Cyclodina whitakeri	*Oligosoma whitakeri*	2-4	Chapple et al. (2008b, 2009)
Leiolopisma acrinasum	*Oligosoma acrinasum*	2-4	Chapple et al. (2009)
Leiolopisma chloronoton	*Oligosoma chloronoton*	2-4	Greaves et al. (2007), Chapple et al. (2009)
	Oligosoma aff. *chloronoton* 'West Otago'	2-3	Greaves et al. (2007), Chapple et al. (2009)
Leiolopisma fallai	*Oligosoma fallai*	2-4	Chapple et al. (2009)

Leiolopisma grande	*Oligosoma grande*	1-4	Patterson (1997), Berry & Gleeson (2005), Chapple et al. (2009)
Leiolopisma homalonotum	*Oligosoma homalonotum*	2-4	Chapple et al. (2009)
Leiolopisma infrapunctatum	*Oligosoma infrapunctatum*	2-4	Greaves et al. (2008), Chapple et al. (2009)
	Oligosoma aff. *infrapunctatum* 'Chesterfield'	2-3	Greaves et al. (2008), Chapple et al. (2009)
	Oligosoma aff. *infrapunctatum* 'Southern North Island'	2-3	Greaves et al. (2008), Chapple et al. (2009)
Leiolopisma lineoocellatum	*Oligosoma lineoocellatum*	2-4	Greaves et al. (2007), Chapple et al. (2009)
	Oligosoma aff. *lineoocellatum* 'South Marlborough'	2	Greaves et al. (2007)
	Oligosoma aff. *lineoocellatum* 'Mackenzie Basin'	2-3	Greaves et al. (2007), Chapple et al. (2009)
	Oligosoma aff. *lineoocellatum* 'Central Canterbury'	2	Greaves et al. (2007)
Leiolopisma moco	*Oligosoma moco*	2-4	Hare et al. (2008), Chapple et al. (2009)
Leiolopisma nigriplantare nigriplantare	*Oligosoma nigriplantare*	2-4	Liggins et al. (2008a), Chapple et al. (2009)
Leiolopisma nigriplantare maccanni	*Oligosoma polychroma*	1-4	Daugherty et al. (1990), Patterson & Daugherty (1990), Liggins et al. (2008b), Chapple et al. (2009)
	Oligosoma aff. *polychroma* 'Clade 2'	2	Liggins et al. (2008b)
	Oligosoma aff. *polychroma* 'Clade 3'	2	Liggins et al. (2008b)
	Oligosoma aff. *polychroma* 'Clade 4'	2	Liggins et al. (2008b)
	Oligosoma aff. *polychroma* 'Clade 5'	2	Liggins et al. (2008b), Chapple et al. (2009)

	Oligosoma inconspicuum	1-4	Daugherty et al. (1990), Patterson & Daugherty (1990), Chapple et al. (2009, 2011)
	Oligosoma burganae	2,4	Chapple et al. (2011)
	Oligosoma toka	2,4	Chapple et al. (2011)
	Oligosoma repens	2,4	Chapple et al. (2011)
	Oligosoma longipes	1-4	Patterson (1997), Chapple et al. (2009)
	Oligosoma aff. *longipes* 'Southern'	2-3	Chapple et al. (2009)
	Oligosoma maccanni	1-4	Daugherty et al. (1990), Patterson & Daugherty (1990), O'Neill et al. (2008), Chapple et al. (2009)
	Oligosoma notosaurus	1-4	Daugherty et al. (1990), Patterson & Daugherty (1990), Chapple et al. (2009, 2011)
	Oligosoma stenotis	1-4	Patterson & Daugherty (1994), Chapple et al. (2009)
	Oligosoma microlepis	1-4	Daugherty et al. (1990), Patterson & Daugherty (1990), Hare et al. (2008), Chapple et al. (2009)
Leiolopisma otagense form otagense	*Oligosoma otagense*	1-4	Patterson (1997), Chapple et al. (2009, 2012)
Leiolopisma otagense form waimatense	*Oligosoma waimatense*	1-4	Patterson (1997), Chapple et al. (2009, 2012)
Leiolopisma smithi	*Oligosoma smithi*	2-4	Hare et al. (2008), Chapple et al. (2009)
	Oligosoma smithi 'Three Kings, Te Paki, Western Northland'	2	Hare et al. (2008), Chapple et al. (2009)
Leiolopisma striatum	*Oligosoma striatum*	2-4	Chapple et al. (2009)
Leiolopisma suteri	*Oligosoma suteri*	2-4	Hare et al. (2008), Chapple et al. (2009)

Leiolopisma zelandicum	*Oligosoma zelandicum*	2-4	O'Neill et al. (2008), Chapple et al. (2009)
Leiolopisma lichenigerum	*Oligosoma lichenigerum*	2-3	Chapple et al. (2009)
Not discovered	*Oligosoma judgei*	2,4	Patterson & Bell (2009)
	Oligosoma pikitanga	2-4	Bell & Patterson (2008), Chapple et al. (2009)
	Oligosoma taumakae	1-4	Chapple & Patterson (2007), Chapple et al. (2009)
	Oligosoma tekakahu	2,4	Chapple et al. (2011)
	Oligosoma aff. *longipes* 'Rangitata'	2-4	Chapple et al. (2009)
	Oligosoma aff. *inconspicuum* 'Okuru'	4-5	Chapple et al. (2011)
	Oligosoma 'Whirinaki'	2,4	Hitchmough et al. (2010)

References

Bell TP, Patterson GB, 2008. A rare alpine skink *Oligosoma pikitanga* n. sp. (Reptilia: Scincidae) from Llawrenny Peaks, Fiordland, New Zealand. Zootaxa 1882:57-68.

Berry O, Gleeson DM, 2005. Distinguishing historical fragmentation from a recent population decline- shrinking or pre-shrunk skink from New Zealand? Biological Conservation 123:197-210.

Chapple DG, Patterson GB, 2007. A new skink species (*Oligosoma taumakae* sp. nov.; Reptilia: Scincidae) from the Open Bay Islands, New Zealand. New Zealand Journal of Zoology 34:347-357.

Chapple DG, Patterson GB, Bell T, Daugherty CH, 2008a. Taxonomic revision of the New Zealand Copper Skink (*Cyclodina aenea*; Squamata: Scincidae) species complex, with description of two new species. Journal of Herpetology 42:437-452.

Chapple DG, Daugherty CH, Ritchie PA, 2008b. Comparative phylogeography reveals pre-decline population structure of New Zealand *Cyclodina* (Reptilia: Scincidae) species. Biological Journal of the Linnean Society 95:388-408.

Chapple DG, Patterson GB, Gleeson DM, Daugherty CH, Ritchie PA, 2008c. Taxonomic revision of the marbled skink (*Cyclodina oliveri,* Reptilia: Scincidae) species complex, with a description of a new species. New Zealand Journal of Zoology 35:129-146.

Chapple DG, Ritchie PA, Daugherty CH, 2009. Origin, diversification and systematics of the New Zealand skink fauna (Reptilia: Scincidae). Molecular Phylogenetics and Evolution 52:470-487.

Chapple DG, Bell TP, Chapple SNJ, Miller KA, Daugherty CH, Patterson GB, 2011. Phylogeography and taxonomic revision of the New Zealand cryptic skink (*Oligosoma inconspicuum*; Reptilia: Scincidae) species complex. Zootaxa 2782:1-33.

Chapple DG, Birkett A, Miller KA, Daugherty CH, Gleeson DM, 2012. Phylogeography of the endangered Otago skink, *Oligosoma otagense*: population structure, hybridisation and genetic diversity in captive populations. PLoS ONE 7:e34599.

Daugherty CH, Patterson GB, Thorn CJ, French DC, 1990. Differentiation of the members of the New Zealand *Leiolopisma nigriplantare* species complex (Lacertilia: Scincidae). Herpetological Monographs 4:61-76.

Greaves SNJ, Chapple DG, Gleeson DM, Daugherty CH, Ritchie PA, 2007. Phylogeography of the spotted skink (*Oligosoma lineoocellatum*) and green skink (*O. chloronoton*) species complex (Lacertilia: Scincidae) in New Zealand reveals pre-Pleistocene divergence. Molecular Phylogenetics and Evolution 45:729-739.

Greaves SNJ, Chapple DG, Daugherty CH, Gleeson DM, Ritchie PA, 2008. Genetic divergences pre-date Pleistocene glacial cycles in the New Zealand speckled skink, *Oligosoma infrapunctatum*. Journal of Biogeography 35:853-864.

Hare KM, Daugherty CH, Chapple DG, 2008. Comparative phylogeography of three skink species (*Oligosoma moco*, *O. smithi* and *O. suteri*; Reptilia: Scincidae) in northeastern New Zealand. Molecular Phylogenetics and Evolution 46:303-315.

Hitchmough RA, Hoare JM, Jamieson H, Newman DG, Tocher MD, Anderson PJ, Lettink M, Whitaker AH, 2010. Conservation status of New Zealand reptiles, 2009. New Zealand Journal of Zoology 37:203-224.

Liggins L, Chapple DG, Daugherty CH, Ritchie PA, 2008a. Origin and post-colonization evolution of the Chatham Islands skink (*Oligosoma nigriplantare nigriplantare*). Molecular Ecology 17:3290-3305.

Liggins L, Chapple DG, Daugherty CH, Ritchie PA, 2008b. A SINE of restricted gene flow across the Alpine Fault: phylogeography of the New Zealand common skink (*Oligosoma nigriplantare polychroma*). Molecular Ecology 17:3668-3683.

O'Neill SB, Chapple DG, Daugherty CH, Ritchie PA, 2008. Phylogeography of two New Zealand lizards: McCann's skink (*Oligosoma maccanni*) and the brown skink (*O. zelandicum*). Molecular Phylogenetics and Evolution 48:1168-1177.

Patterson GB, 1997. South Island skinks of the genus *Oligosoma*: description of *O. longipes* n. sp with redescription of *O. otagenase* (McCann) and *O. waimatense* (McCann). Journal of the Royal Society of New Zealand 27:439-450.

Patterson GB, Bell TP, 2009. The Barrier skink *Oligosoma judgei* n. sp. (Reptilia: Scincidae) from the Darran and Takitimu Mountains, South Island, New Zealand. Zootaxa 2271:43-56.

Patterson GB, Daugherty CH, 1990. Four new species and one new subspecies of skinks, genus *Leiolopisma* (Reptilia: Lacertilia: Scincidae) from New Zealand. Journal of the Royal Society of New Zealand 20:65-84.

Patterson GB, Daugherty CH, 1994. *Leiolopisma stenotis*, n. sp. (Reptilia: Lacertilia: Scincidae) from Stewart Island, New Zealand. Journal of the Royal Society of New Zealand 20:65-84.

Vos ME, 1988. A biochemical, morphological and phylogenetic review of the genus *Cyclodina*. Masters Thesis, Victoria University of Wellington, New Zealand.
