# Supplementary material for: A Retrospective Approach to Testing the DNA Barcoding Method
Source: PLoS One. 2013 Nov 11;8(11):e77882. doi: 10.1371/journal.pone.0077882 (PMC3823873; doi:10.1371/journal.pone.0077882)
Supplement: Table S2 — Locality data, museum voucher specimen information, and GenBank accession numbers for the New Zealand skink samples used in this study. Samples with CD or FT codes were obtained from the National Frozen Tissue Collection (NFTC) housed at Victoria University of Wellington, New Zealand (the associated voucher specimens are now housed at Te Papa). Samples with RE codes were obtained from Te Papa, National Museum of New Zealand, Wellington (S codes refer to specimens from the former Ecology Division collection, now housed at Te Papa). Samples with ABTC (Australian Biological Tissue Collection) codes were obtained from the South Australian Museum. Samples with NR and EBU codes were obtained from the Australian Museum. Asterisks indicate the exemplar specimens. (PDF) [file pone.0077882.s002.pdf]

Table S2. Locality data, museum voucher specimen information, and GenBank accession numbers for the New Zealand skink samples used in this study. Samples with CD or FT codes were obtained from the National Frozen Tissue Collection (NFTC) housed at Victoria University of Wellington, New Zealand (the associated voucher specimens are now housed at Te Papa). Samples with RE codes were obtained from Te Papa, National Museum of New Zealand, Wellington (S codes refer to specimens from the former Ecology Division collection, now housed at Te Papa). Samples with ABTC (Australian Biological Tissue Collection) codes were obtained from the South Australian Museum. Samples with NR and EBU codes were obtained from the Australian Museum. Asterisks indicate the exemplar specimens.

Species	Sample Code	Museum Voucher	Collection Locality	GenBank Accession No.
O. acrinasum	OAC1	CD826	Fiordland	KC349640
	OAC3*	RE1839	Five Fingers Peninsula, Fiordland	KC349641
O. aeneum	CAE1	FT171	Korapuki Island, Mercury Islands	KC349568
	CAE2*	FT5253	Pukerua Bay	KC349571
	CAE4	FT184	Pukerua Bay	KC349573
	CAE6	FT189	Devonport, Auckland	KC349575
	CAE7	FT190	Devonport, Auckland	KC349576
	CAE8	FT611	Red Mercury Island, Mercury Islands	KC349577
	CAE11	CD1962	Matiu-Somes Island	KC349569
	CAE12	FT6535	Seatoun	KC349570
	CAE25	RE4610 (S970)	Mana Island	KC349572
	CAE43	RE4876 (S1239)	Red Rock, Karaka Bay, Great Barrier Island	KC349574

O. alani	CAL1*	FT145	Green Island, Mercury Islands	KC349579
	CAL2	FT3016	Matapia Island	KC349580
	CAL3	FT144	Green Island, Mercury Islands	KC349581
	CAL4	FT146	Middle Island, Mercury Islands	KC349582
	CAL5	FT147	Middle Island, Mercury Islands	KC349583
	CAL7	RE4660 (S1021)	Castle Rock, 8 miles E of Whitianga	KC349584
O. chloronoton	OCH1	FT555	Codfish Island, Stewart Island	KC349644
	OCH9	CD382	Emerald Creek	KC349651
	OCH10	CD424	Big Island	KC349645
	OCH11*	CD1280	Tiwai Point	KC349646
	OCH18	FT3632	Catlins	KC349649
O. aff. *choronoton* 'West Otago'	OCH2*	CD847	Tara Hills, Omarama	KC349650
	OCH12	CD1294	Lindis Pass	KC349647
	OCH13	CD1904	Gorge Burn, Eyre Mountains	KC349648
O. fallai	OFA1*	FT597	Great Island, Three Kings Islands	KC349652
	OFA2	FT598	Great Island, Three Kings Islands	KC349653
O. grande	OGR1*	CD1055	Central Otago	KC349654
O. hardyi	PKS1*	CD1036	Aorangi Island, Poor Knights Island	KC349848
	PKS2	CD1037	Aorangi Island, Poor Knights Island	KC349849
O. homalonotum	OHO1*	FT6290	Shoal Bay, Great Barrier Island	KC349655
	OHO2	FT6291	Tryphena, Great Barrier Island	KC349656
O. inconspicuum	OIN1*	CD1101	Gorge Burn, Eyre Mountains	KC349691
	OIN2	CD421	Macraes Flat	KC349692

	OIN3	—	Macraes Flat	KC349693
	BBS1	FT3783	Awarua Point, Big Bay, Westland	KC349552
	BBS2	FT3786	Mouth of Mackenzie River, Big Bay, Westland	KC349560
	BBS3	FT3784	Awarua Point, Big Bay, Westland	KC349562
	BBS4	FT3785	Awarua Point, Big Bay, Westland	KC349563
	BBS5	FT3787	Mouth of Mackenzie River, Big Bay, Westland	KC349564
	BBS6	FT3788	Mouth of Mackenzie River, Big Bay, Westland	KC349565
	BBS7	FT3789	mid-point Mckenzie River, Big Bay, Westland	KC349566
	BBS9	FT3791	mid-point Mckenzie River, Big Bay, Westland	KC349567
	BBS10	FT3792	Big Bay, Westland	KC349553
	BBS11	FT3793	Big Bay, Westland	KC349554
	BBS14	FT3032	Awarua Point, Big Bay, Westland	KC349555
	BBS15	FT3033	Awarua Point, Big Bay, Westland	KC349556
	BBS17	FT3813	Barn Bay, West Coast	KC349557
	BBS18	FT7652	Cascade Plateau	KC349558
	BBS19	FT7653	Cascade Plateau	KC349559
	BBS20	FT7654	Cascade Plateau	KC349561
O. infrapunctatum	OIF1*	CD545	Stephens Island	KC349657
	OIF2	FT3749	Cobden Beach	KC349666
	OIF5	FT6272	Denniston	KC349685
	OIF10	CD531	Stephens Island	KC349658
	OIF11	FT3395	Granity	KC349659
	OIF12	FT3481	Hokitika	KC349660

	OIF15	FT3758	Birchfield Beach	KC349662
	OIF17	FT6270	Brown Hill, Heaphy Track	KC349664
	OIF19	RE 5235	Hokitika	KC349665
	OIF22	RE 5247	Rotorua	KC349667
	OIF25	FT3023	Whale Island	KC349669
	OIF26	FT3024	Whale Island	KC349670
	OIF28	FT3740	Hokitika Cemetery	KC349671
	OIF29	FT3741	Hokitika Cemetery	KC349672
	OIF30	FT3742	Hokitika Cemetery	KC349674
	OIF31	FT3809	Orowaitai Lagoon	KC349675
	OIF33	FT3476	Hokitika	KC349676
	OIF34	FT3767	Oparara Rd, Karamea	KC349677
	OIF35	FT3768	Oparara Rd, Karamea	KC349678
	OIF39	FT3795	Costello Hill, Pakahi Charleston	KC349681
	OIF40	FT3799	Kaniere Rd, Hokitika	KC349683
	OIF41	FT3800	Kaniere Rd, Hokitika	KC349684
	PAP1	FT3815	Paparoa Ranges	KC349847
O. aff. *infrapunctatum* ‘Chesterfield’	OIF3*	FT3214	Chesterfield	KC349673
	OIF4	FT3215	Chesterfield	KC349682
	OIF13	FT3745	Alborn Mine	KC349661
	OIF37	FT3770	Chesterfield	KC349679
	OIF38	FT3771	Chesterfield	KC349680

O. aff. *infrapunctatum* ‘Southern North Island’	OIF6*	—	Waiinu Beach	KC349687
	OIF7	—	Waiinu Beach	KC349688
	OIF8	—	Waiinu Beach	KC349689
	OIF9	—	Waiinu Beach	KC349690
	OIF16	FT6269	Waiinu Beach	KC349663
	OIF24	FT3004	Westport	KC349668
	OIF57	—	Castlecliff	KC349686
O. levidensum	CAE9*	FT3729	Kauri Bush, Pandora Track, Northland	KC349578
O. lineoocellatum	OLI6	CD430	Ward Island	KC349705
	OLI7	CD431	Ward Island	KC349706
	OLI8*	CD463	Cape Turakirae	KC349707
	OLI19	—	Lake Station	KC349699
	OLI28	CD601	Stephens Island	KC349701
	OLI29	CD797	Aniseed Valley	KC349702
	OLI30	FT239	North Brother Island	KC349703
O. aff. *lineoocellatum* ‘South Marlborough’	OLI1*	FT302	Jack Taylor's Farm, Ward, South Island	KC349694
O. aff. *lineoocellatum* ‘Mackenzie Basin’	OLI2*	FT3112	Tekapo	KC349700
	OLI10	CD1217	Tekapo	KC349695
	OLI14	FT3211	Tekapo	KC349697
	OLI15	FT3212	Tekapo	KC349698

O. aff. *lineoocellatum* 'Central Canterbury'	OLI4	—	Birdlings Flat	KC349704
	OLI11*	CD1064	Ashburton	KC349696
O. longipes	OLO1*	FT161	Clarence River, Lake Tennyson	KC349708
	OLO4	FT160	Clarence River, Lake Tennyson	KC349711
O. aff. *longipes* 'Southern'	OLO2	—	Mt Harper	KC349709
	OLO3	—	Mt Harper	KC349710
	OLO6*	CD1039	Mt Hay, Lake Tekapo	KC349712
	OLO7	CD803	Mount Hay Station, Edwards Stream, Lake Tekapo	KC349713
O. aff. *longipes* 'Rangitata'	RAN1*	—	Mt Harper	KC349850
	RAN2	—	Mt Harper	KC349851
O. maccanni	OMA1	CD930	Nevis Range, central Otago	KC349714
	OMA2*	CD1261	Puketoi, Otago	KC349722
	OMA3	FT3213	Garston, Southland/Westland	KC349732
	OMA4	FT3039	Garston, Southland/Westland	KC349733
	OMA5	—	Alexandra, central Otago	KC349734
	OMA6	—	Alexandra, central Otago	KC349735
	OMA7	—	Macraes Flat, Otago	KC349736
	OMA8	—	Macraes Flat, Otago	KC349737
	OMA9	CD956	Edwards Creek, Canterbury	KC349738
	OMA10	CD804	Mount Hay Station, Canterbury	KC349715
	OMA11	CD615	Birdlings Flat, Canterbury	KC349716
	OMA12	CD1254	Gordon Peak, Knobby Range, Otago	KC349717

	OMA13	CD1119	Otematata, Canterbury	KC349718
	OMA14	CD1106	Gorge Burn, Eyre Mountains, Southland	KC349719
	OMA15	CD778	Burgan Stream Hut, Otago	KC349720
	OMA16	CD1255	Hills Creek, Otago	KC349721
	OMA20	CD635	Mount John Station, Canterbury	KC349723
	OMA21	CD634	Simons Hill, Canterbury	KC349724
	OMA22	CD627	Mount Mary, Canterbury	KC349725
	OMA23	CD626	Taieri Ridge, Otago	KC349726
	OMA24	CD567	Conroys Dam, Otago	KC349727
	OMA25	CD428	Remarkables, Otago	KC349728
	OMA26	CD426	10 km North of Millers Flat, Otago	KC349729
	OMA27	CD425	Frankton, Otago	KC349730
	OMA29	CD10	Hokonui Hills, Southland	KC349731
	ONP30	CD1293	Lindis Pass	KC349765
	ONP38	CD1044	Mt Hay, Lake Tekapo	KC349772
O. macgregori	CMA1*	FT3	Mana Island	KC349585
	CMA2	FT1095	Mana Island	KC349586
O. microlepis	OMI1*	CD1299	Taihape	KC349739
	OMI2	FT3730	Mokimokonui River, Southern Urewera	KC349740
O. moco	OMO1	FT156	Stanley Island, Mercury Islands	KC349741
	OMO2	CD1031	Aorangi Island, Poor Knights Islands	KC349742
	OMO3	CD848	Lady Alice Island, Hen & Chickens Islands	KC349743
	OMO4*	FT167	Korapuki Island, Mercury Islands	KC349744

	OMO5	FT296	Middle Island, Mercury Islands	KC349745
	OMO6	FT2903	Cuvier Island	KC349746
	OMO7	RE5161 (S1526)	Whangapoua Creek, Great Barrier Island	KC349747
O. nigriplantare	ONN8*	FT3292	Middle Sister Island, Chatham Islands	KC349748
	ONN9	FT3293	Middle Sister Island, Chatham Islands	KC349749
O. notosaurus	ONT1	FT575	Magog, Stewart Island	KC349795
	ONT2*	CD1089	Arena Ridge, Stewart Island	KC349796
O. oliveri	COL1*	CD1034	Aorangi Island, Poor Knights Islands	KC349587
	COL2	CD1035	Aorangi Island, Poor Knights Islands	KC349593
	COL3	FT137	Green Island, Mercury Islands	KC349594
	COL4	FT572	Ruamahuanui, Aldermen Islands	KC349595
	COL6	CD819	Middle Island, Mercury Islands	KC349596
	COL7	CD820	Middle Island, Mercury Islands	KC349597
	COL8	CD821	Middle Island, Mercury Islands	KC349598
	COL9	FT138	Green Island, Mercury Islands	KC349599
	COL10	FT139	Green Island, Mercury Islands	KC349588
	COL11	FT142	Middle Island, Mercury Islands	KC349589
	COL12	FT143	Middle Island, Mercury Islands	KC349590
O. ornatum	COR1	FT188	Devonport, Auckland	KC349600
	COR2*	FT3733	Botanic Gardens, Wellington	KC349602
	COR4	FT583	Hen Island, Hen & Chickens	KC349604
	COR5	FT594	Lady Alice Island, Hen & Chickens	KC349605
	COR6	FT3113	Matapia Island	KC349606

	COR15	RE4732 (S1093)	Kapiti Island	KC349601
O. aff. *ornatum* ‘Poor Knights Islands’	COR28*	RE1626	Aorangi Island, Poor Knights Island	KC349603
O. otagense	OOT2*	—	Macraes Flat	KC349800
	OOT11	—	Alistairs Gully, Macraes Flat	KC349797
	OOT12	—	Lindis Pass	KC349798
	OOT13	—	Lindis Pass	KC349799
O. pikitanga	SVS1*	RE5315 (FT7648)	Sinbad Gully, Llawrenny Peaks, Fiordland	KC349852
O. polychroma	ONP1*	FT5252	Pukerua Bay, Wellington	KC349750
	ONP13	CD792	Aniseed Valley, Canterbury	KC349754
	ONP31	FT533	Makara, Wellington	KC349766
	ONP33	RE.5368 (FT3774)	Mania Valley, Nelson	KC349767
	ONP37	CD496	Motueka, Nelson	KC349771
	ONP40	CD1065	Mitchell Bush, Manawatu-Wanganui	KC349775
	ONP45	FT6486	Palmer Head, Wellington	KC349778
	ONP50	CD1956	Matiu-Somes Island, Wellington	KC349783
	ONP51	FT3732	Springs Junction, West Coast	KC349784
	ONP52	FT3000	St Amaud, Nelson	KC349785
	ONP53	CD110	Stephens Island, Nelson	KC349786
	ONP54	FT3015	Tahuranui, Nelson	KC349787
	ONP60	FT3693	Waipakihi River, Manawatu-Wanganui	KC349790
	ONP61	FT300	Wairau River Mouth, Marlborough	KC349791
	ONP62	FT3694	Wakemans Clearing, Manawatu-Wanganui	KC349792

	ONP63	CD265	Ward Island, Wellington	KC349793
	GVS1	FT4796	Kangaroo Creek, West Coast	KC349620
	GVS2	RE.5398 (FT3804)	Blaketown Beach, West Coast	KC349621
	GVS3	RE.5391 (FT3797)	Kangaroo Creek, West Coast	KC349622
	GVS4	RE.5399 (FT3805)	Blaketown Beach, West Coast	KC349623
	GVS5	RE.5400 (FT3806)	Blaketown Beach, West Coast	KC349624
	GVS6	RE.5501 (FT3807)	Blaketown Beach, West Coast	KC349625
	GVS7	RE.5502 (FT3808)	Blaketown Beach, West Coast	KC349626
	GVS8	RE.5392 (FT3798)	Mawheraiti Railway, West Coast	KC349627
O. aff. *polychroma* 'Clade 3'	ONP15*	FT162	Clarence River, Canterbury	KC349756
O. aff. *polychroma* 'Clade 4'	ONP27*	CD2024	Lake Heron, Canterbury	KC349762
	ONP29	FT2996	The Poplars, Canterbury	KC349764
	ONP36	FT2998	Montrose, Canterbury	KC349770
	ONP43	RE.5369 (FT3775)	Oneone pakihi, West Coast	KC349776
O. aff. *polychroma* 'Clade 5'	ONP2	CD618	Birdlings Flat, Canterbury	KC349760
	ONP4	FT3219	Tiwai Point, Southland	KC349774
	ONP5	FT3041	Tiwai Point, Southland	KC349782
	ONP6*	—	Macraes Flat, Otago	KC349789
	ONP8	FT3042	Tiwai Point, Southland	KC349794
	ONP10	FT3040	Tiwai Point, Southland	KC349751
	ONP11	FT3217	Tiwai Point, Southland	KC349752
	ONP12	FT3218	Tiwai Point, Southland	KC349753
	ONP14	FT298	Banks Peninsula, Canterbury	KC349755

	ONP16	FT550	Codfish Island, Southland	KC349757
	ONP18	CD950	Edwards Creek, Canterbury	KC349758
	ONP19	CD1113	Eyre Mountains, Southland	KC349759
	ONP25	FT538	Lake Hawea, Otago	KC349761
	ONP28	CD1004	Lake Pukaki, Canterbury	KC349763
	ONP34	CD1256	Manorburn, Southland	KC349768
	ONP35	FT614	Stewart Island, Southland	KC349769
	ONP39	CD1042	Mount Mary, Canterbury	KC349773
	ONP44	CD1116	Otematata, Otago	KC349777
	ONP47	CD1257	Puketoi, Otago	KC349779
	ONP48	CD780	Rock and Pillar Range, Otago	KC349780
	ONP49	FT2067	Wainono, Canterbury	KC349781
	ONP58	CD2126	Twizel, Canterbury	KC349788
O. smithi	OSM1	FT166	Middle Island, Mercury Islands	KC349801
	OSM2*	FT193	Ocean Beach, Whangarei	KC349810
	OSM4	CD1029	Sugarloaf Island, Poor Knights Islands	KC349815
	OSM5	CD1030	Aorangi Island, Poor Knights Islands	KC349816
	OSM6	CD1074	Little Barrier Island	KC349817
	OSM7	FT140	Green Island, Mercury Islands	KC349818
	OSM8	FT154	Stanley Island, Mercury Islands	KC349819
	OSM9	FT168	Korapuki Island, Mercury Islands	KC349820
	OSM10	FT539	Moutoki Island, Bay of Plenty	KC349802
	OSM12	FT549	Karewa Island, Bay of Plenty	KC349803

	OSM13	FT569	Raumahuea-iti, Alderman Islands	KC349804
	OSM15	FT3021	Matakana Island, Bay of Plenty	KC349806
	OSM16	FT3299	Waiomu Stream mouth, north of Thames, Coromandel Peninsula	KC349807
	OSM17	RE5172 (S1537)	Tucks Bay, Coromandel Peninsula	KC349808
	OSM19	RE5024 (S1387)	Marotiri Island, Hen & Chickens Islands	KC349809
	OSM32	RE4822 (S1184)	Lizard Isle, Mokohinau Islands	KC349813
	OSM37	RE4694 (S1055)	Hongiora, Alderman Islands	KC349814
O. aff. *smithi* 'Three Kings, Te Paki, Western Northland'	OSM14	FT601	Great Island, Three Kings Islands	KC349805
	OSM31*	RE4833 (S1195)	Muriwai Beach, 30 km NW of Auckland	KC349812
O. stenotis	OST1*	FT2	Mt Anglem, Stewart Island	KC349823
	OST2	FT289	Table Hill, Stewart Island	KC349824
O. striatum	OSR1*	FT3301	Waipuku, Taranaki	KC349821
	OSR2	FT3296	Little Barrier Island	KC349822
O. suteri	OSU1	FT148	Green Island, Mercury Islands	KC349825
	OSU2	FT602	Great Island, Three Kings Islands	KC349831
	OSU3	CD1027	Aorangi Island, Poor Knights Islands	KC349832
	OSU4*	FT158	Middle Island, Mercury Islands	KC349833
	OSU5	FT570	Rumamahuea-iti, Alderman Islands	KC349834
	OSU6	FT578	Hen Island, Hen & Chickens Islands	KC349835
	OSU7	FT624	One, NW Hen & Chickens Islands	KC349836
	OSU8	FT2493	Ware Ware Island, NW Hen & Chickens Islands	KC349837

	OSU9	FT2496	Muriwhenua Island, Hen & Chickens Islands	KC349838
	OSU10	FT5494	Green Island, Mercury Islands	KC349826
	OSU11	RE4819 (S1181)	Burgess Island, Mokohinau Islands	KC349827
	OSU14	RE4716 (S1077)	Flat Island, off Ohinau Island, Coromandel Peninsula	KC349828
	OSU15	RE4587 (S947)	Taupo Bay, Whangaroa Bay, Northland	KC349829
	OSU16	RE4583 (S943)	Tapotupotu Bay, Northland	KC349830
	OSM3	CD850	Muriwhenua Island, Hen & Chickens Islands	KC349811
O. taumakae	OBI2*	RE5237	Taumaka, Open Bay Islands	KC349642
	OBI3	FT311	Taumaka, Open Bay Islands	KC349643
O. tekakahu	TEK1*	FT7650	Chalky Island, Fiordland	KC349853
O. townsi	MOS1	RE5917 (FT182)	Mokohinau Island	KC349630
	MOS2*	RE5918 (FT617)	Pupuha Island, Hen & Chicken Islands	KC349632
	MOS3	RE5916 (FT181)	Mokohinau Island	KC349633
	MOS4	RE5921 (FT623)	Pupuha Island, Hen & Chicken Islands	KC349634
	MOS5	RE5919 (FT619)	Muriwhenua, Hen & Chicken Island	KC349635
	MOS6	RE5920 (FT621)	Muriwhenua, Hen & Chicken Island	KC349636
	MOS7	RE5922 (FT625)	Muriwhenua, Hen & Chicken Island	KC349637
	MOS8	RE5923 (FT626)	Unknown locality, Hen & Chickens Group	KC349638
	MOS9	RE4800 (S1162)	Stack ‘H’ W of Trig (Atihau) Island, Mokohinau Islands	KC349639
	MOS10	RE4801 (S1163)	Stack ‘H’ W of Trig (Atihau) Island, Mokohinau Islands	KC349631

	COL14	—	Ahuriri Stream, Great Barrier Island	KC349591
	COL15	—	Ahuriri Stream, Great Barrier Island	KC349592
O. waimatense	OWA2	CD1209	Wairau River, Marlborough	KC349839
	OWA5	CD1214	Little Mt Ida, Otago	KC349840
	OWA6	FT3011	Rag and Famish Strm Valley, Marlborough	KC349841
	OWA7*	FT3012	Black Jacks Island, Lake Benmore, Otago	KC349842
O. whitakeri	CWH1	CD949	Pukerua Bay	KC349607
	CWH2*	FT294	Middle Island, Mercury Islands	KC349608
	CWH3	CD809	Middle Island, Mercury Islands	KC349609
	CWH4	CD822	Middle Island, Mercury Islands	KC349610
	CWH5	FT186	Pukerua Bay	KC349611
	CWH6	FT531	Middle Island, Mercury Islands	KC349612
	CWH7	FT297	Castle Island	KC349613
O. zelandicum	OZE2	FT6516	Pukerua Bay, Wellington	KC349843
	OZE4*	CD331	Maud Island, Nelson/Marlborough	KC349844
	OZE7	RE5337 (FT3743)	Gentle Annie, West Coast	KC349845
	OZE9	FT6525	Johnsonville, Wellington	KC349846
O. lichenigerum	LIC1*	ABTC68934 (SAMAR52030)	Blackburn Island, Lord Howe Island	KC349628
	LIC2	ABTC58889 (SAMAR52161)	Blackburn Island, Lord Howe Island	KC349629
Nannoscincus mariei	EUG1	NR9808	New Caledonia	KC349614
Marmorosphax tricolor	EUG2	NR9800	New Caledonia	KC349616

Caledoniscincus austrocaledonicus	EUG3	EBU3522	New Caledonia	KC349617
Lioscincus tillieri	EUG5	NR3617	Mt Mou, New Caledonia	KC349618
Lampropholis guichenoti	EUG7	NR2639	Australia	KC349619
Morethia adelaidensis	EUG10	NR8560	Australia	KC349615
