# Supplementary material for: A Retrospective Approach to Testing the DNA Barcoding Method
Source: PLoS One. 2013 Nov 11;8(11):e77882. doi: 10.1371/journal.pone.0077882 (PMC3823873; doi:10.1371/journal.pone.0077882)
Supplement: Table S3 — Oligonucleotide primers used in this study to amplify and sequence COI in New Zealand skinks. (PDF) [file pone.0077882.s003.pdf]

Table S3. Oligonucleotide primers used in this study to amplify and sequence COI in New Zealand skinks.

Gene	Primer Name	Sequence (5'-3')	5' Position	Source
COI	SkinkCOI-F1	TTAACAGCTAARCACCCAATCCAGC	5093	This study
	SkinkCOI-F3	AATCGTTGATTCTTCTCAACCAACC	5297	This study
	SkinkCOI-R2	GGAAGGATAAGAATGTAGACTTCTGGGTG	6005	This study
	COIe	CCAGAGATTAGAGGGAATCAGTG	6470	Palumbi (1996)

Values in '5' position' refer to the position of the 5' position in the complete *Plestiodon egregius* mtDNA sequence (Kumazawa & Nishida 1999).

References

Kumazawa Y, Nishida M, 1999. Complete mitochondrial DNA sequences of the green turtle and blue-tailed mole skink: statistical evidence for Archosaurian affinity of turtles. Molecular Biology and Evolution 16:784-792.

Palumbi SR, 1996. Nucleic Acids II: The polymerase chain reaction. In: Hillis DM, Moritz C, Mable MK, editors. Molecular Systematics Sunderland: Sinauer Associates. p. 205-247.
