# Supplementary material for: A Retrospective Approach to Testing the DNA Barcoding Method
Source: PLoS One. 2013 Nov 11;8(11):e77882. doi: 10.1371/journal.pone.0077882 (PMC3823873; doi:10.1371/journal.pone.0077882)
Supplement: Table S4 — Query identification criteria for the NJ-based and matching methods (modified from Meier et al. 2006). (PDF) [file pone.0077882.s004.pdf]

Table S4. Query identification criteria for the Neighbour Joining-based and matching methods (modified from Meier et al. 2006).

Method	***Identification Outcome***		
	Successful	Ambiguous/Unidentified	Failure/Mis-identification
**Neighbour Joining-based, revised criteria**	Query in polytomy with conspecifics OR Query at least one node into a clade exclusively consisting of conspecifics	Query without a conspecific sequence in the dataset OR Query sister group to conspecifics OR Query in polytomy with at least one conspecific and one allospecific sequence	Query at least one node into an allospecific clade OR Query in polytomy with only allospecific sequences
**"Best Match"**	Sequence(s) with smallest distance to query all conspecific	Sequence(s) with smallest distance to query a mixture of allo- and conspecific sequences	All sequence(s) with smallest distance to query allospecific
**"Best Close Match"**	Sequence(s) with smallest distance to query all conspecific and within the 95th percentile of all intraspecific distances	Sequence(s) with smallest distance to query a mixture of allo- and conspecific sequences and within the 95th percentile of all infraspecific distances OR No match within 95th percentile of all infraspecific distances	All sequence(s) with smallest distance to query allospecific and within 95th percentile of all infraspecific distances
**"All Species Barcode"**	As in "Best Match", but with all conspecific sequences topping the list of best matches	As in "Best Match", but with at least one allospecific sequence being more similar to query than the least similar conspecific sequence	Query with conspecific sequence in dataset is more similar to all sequences from another species

Reference

Meier R, Shiyang K, Vaidya G, Ng PKL, 2006. DNA barcoding and taxonomy in Diptera: a tale of high intraspecific variability and low identification. Systematic Biology 55:715-726.
