# Supplementary material for: A Retrospective Approach to Testing the DNA Barcoding Method
Source: PLoS One. 2013 Nov 11;8(11):e77882. doi: 10.1371/journal.pone.0077882 (PMC3823873; doi:10.1371/journal.pone.0077882)
Supplement: Table S5 — Number of samples and geographic localities used in the DNA barcoding study of New Zealand skinks based on the 1977 taxonomy. (See Tables S1 and S2 for additional details.) The level (mean ± standard error [SE], and range) of intraspecific K2P genetic distances is shown for each New Zealand skink species. The sample codes (see Table S2) for the new discoveries since 1977 are indicated. (PDF) [file pone.0077882.s005.pdf]

Table S5. Number of samples and geographic localities used in the DNA barcoding study of New Zealand skinks based on the 1977 taxonomy (see Tables S1 and S2 for additional details). The level (mean ± standard error [SE], and range) of intraspecific K2P genetic distances is shown for each New Zealand skink species. The sample codes (see Table S2) for the new discoveries since 1977 are indicated.

**Species**	**No. samples**	**No. geographic localities**	**Intraspecific genetic distance**	
			Mean ± SE	Range
Cyclodina aenea	13	10	6.57 ± 0.62	0 – 13.9
Cyclodina alani	6	4	1.83 ± 0.31	0 – 4.3
Cyclodina macgregori	2	1	1.00 ± 0.36	1.0
Cyclodina oliveri	23	10	5.73 ± 0.06	0 – 10.4
Cyclodina ornata	7	7	4.47 ± 0.53	1.4 – 7.7
Cyclodina whitakeri	7	3	2.3 ± 0.11	0 – 0.4
Leiolopisma acrinasum	2	2	0.57 ± 0.26	0.6
Leiolopisma chloronoton	8	8	8.20 ± 0.76	1.6 – 11.6
Leiolopisma fallai	2	1	0	0
Leiolopisma grande	1	1	—	—
Leiolopisma homalonotum	2	2	0	0
Leiolopisma infrapunctatum	28	14	3.24 ± 0.38	0 – 7.1
Leiolopisma lineoocellatum	14	10	7.47 ± 0.07	0 – 12.0

Leiolopisma moco	7	7	1.93 ± 0.03	0.1 – 4.7
Leiolopisma nigriplantare nigriplantare	2	1	0.28 ± 0.18	0.3
Leiolopisma nigriplantare maccanni	82	66	13.31 ± 0.94	0 – 21.3
Leiolopisma otagense form otagense	4	2	3.93 ± 0.66	0 – 6.0
Leiolopisma otagense form waimatense	4	4	7.37 ± 0.89	0 – 11.0
Leiolopisma smithi	19	19	2.63 ± 0.32	0 – 7.1
Leiolopisma striatum	2	2	0.42 ± 0.24	0.4
Leiolopisma suteri	15	13	0.12 ± 0.07	0 – 0.4
Leiolopisma zelandicum	4	4	1.22 ± 0.33	0.6- 1.9
Leiolopisma lichenigerum	2	1	0.14 ± 0.13	0.1
**New Discoveries**				
Oligosoma pikitanga (SVS1)				
Oligosoma taumakae (OBI2-3)				
Oligosoma tekakahu (TEK1)				
Oligosoma aff. *infrapunctatum* 'Chesterfield' (OIF3-4, 13, 37-38)				
Oligosoma aff *longipes* 'Rangitata' (RAN1-2)				
**Suspected New Species- Not Validated**				
Oligosoma aff *inconspicuum* 'Big Bay' (BBS1-7,9-11, 14-15)				
Oligosoma aff *inconspicuum* 'Barn Bay' (BBS17)				
Oligosoma aff *inconspicuum* 'Cascade Plateau'				

(BBS18-20)
Oligosoma aff *infrapunctatum* ‘Paparoa’ (PAP1)
Oligosoma aff *infrapunctatum* ‘Denniston’ (OIF5)
Oligosoma aff *maccanni* ‘Garston’ (OMA3-4)
Oligosoma aff *polychroma* ‘Seaward Moss’ (ONP4)
Oligosoma aff *polychroma* ‘Grey Valley’ (GVS1-8)
