# Supplementary material for: A Retrospective Approach to Testing the DNA Barcoding Method
Source: PLoS One. 2013 Nov 11;8(11):e77882. doi: 10.1371/journal.pone.0077882 (PMC3823873; doi:10.1371/journal.pone.0077882)
Supplement: Table S6 — Number of samples and geographic localities used in the DNA barcoding study of New Zealand skinks (genus Oligosoma). (See Table S2 for additional details.) The level (mean ± standard error [SE], and range) of intraspecific K2P genetic distances in each New Zealand skink species. The taxonomy follows the current New Zealand Threat Classification listing (Hitchmough et al. 2010). (PDF) [file pone.0077882.s006.pdf]

Table S6. Number of samples and geographic localities used in the DNA barcoding study of New Zealand skinks (genus *Oligosoma*) (see Table S2 for additional details). The level (mean ± standard error [SE], and range) of intraspecific K2P genetic distances in each New Zealand skink species. The taxonomy follows the current New Zealand Threat Classification listing (Hitchmough et al. 2010).

**Species**	**No. samples**	**No. geographic localities**	**Intraspecific genetic distance**	
			Mean ± SE	Range
Oligosoma acrinasum	2	2	0.57 ± 0.26	0.6
Oligosoma aeneum	10	8	2.29 ± 0.36	0 – 5.0
Oligosoma alani	6	4	1.83 ± 0.31	0 – 4.3
Oligosoma chloronoton	5	5	6.88 ± 0.74	1.6 – 10.7
Oligosoma aff. *choronoton* ‘West Otago’	3	3	1.82 ± 0.04	1.0 – 2.5
Oligosoma fallai	2	1	0	0
Oligosoma grande	1	1	—	—
Oligosoma hardyi	2	1	0.28 ± 0.19	0.3
Oligosoma homalonotum	2	2	0	0
Oligosoma inconspicuum	19	6	2.65 ± 0.35	0 – 5.3
Oligosoma infrapunctatum	23	13	2.01 ± 0.27	0 – 5.9
Oligosoma aff. *infrapunctatum* ‘Chesterfield’	5	2	0.69 ± 0.21	0 – 1.7
Oligosoma aff. *infrapunctatum* ‘Southern North Island’	7	3	0.99 ± 0.02	0 – 2.9

Oligosoma levidensum	1	1	—	—
Oligosoma lineoocellatum	7	6	4.44 ± 0.58	0 – 6.3
Oligosoma aff. *lineoocellatum* ‘South Marlborough’	1	1	—	—
Oligosoma aff. *lineoocellatum* ‘Mackenzie Basin’	4	1	0.07 ± 0.07	0 – 0.1
Oligosoma aff. *lineoocellatum* ‘Central Canterbury’	2	2	1.14 ± 0.38	1.1
Oligosoma longipes	2	1	0	0
Oligosoma aff. *longipes* ‘Southern’	4	2	1.64 ± 0.39	0 – 2.5
Oligosoma aff. *longipes* ‘Rangitata’	2	1	0	0
Oligosoma maccanni	27	23	4.54 ± 0.47	0 – 7.1
Oligosoma macgregori	2	1	1.00 ± 0.36	1.0
Oligosoma microlepis	2	2	0.28 ± 0.02	0.3
Oligosoma moco	7	7	1.93 ± 0.03	0.1 – 4.7
Oligosoma nigriplantare	2	1	0.28 ± 0.18	0.3
Oligosoma notosaurus	2	2	1.29 ± 0.04	1.3
Oligosoma oliveri	11	4	1.01 ± 0.22	0 – 2.2
Oligosoma ornatum	6	6	3.36 ± 0.46	1.4 – 4.8
Oligosoma aff. *ornatum* ‘Poor Knights Islands’	1	1	—	—
Oligosoma otagense	4	2	3.93 ± 0.66	0 – 6.0
Oligosoma pikitanga	1	1	—	—
Oligosoma polychroma	24	19	2.63 ± 0.36	0 – 5.5
Oligosoma aff. *polychroma* ‘Clade 3’	1	1	—	—
Oligosoma aff. *polychroma* ‘Clade 4’	4	4	5.06 ± 0.61	3.7 – 5.8

Oligosoma aff. *polychroma* ‘Clade 5’	22	17	2.19 ± 0.32	0 – 4.6
Oligosoma smithi	17	17	1.62 ± 0.25	0 – 4.1
Oligosoma aff. *smithi* ‘Three Kings, Te Paki, Western Northland’	2	2	3.8 ± 0.75	3.8
Oligosoma stenotis	2	2	2.16 ± 0.54	2.2
Oligosoma striatum	2	2	0.42 ± 0.24	0.4
Oligosoma suteri	15	13	0.12 ± 0.07	0 – 0.4
Oligosoma taumakae	2	1	0	0
Oligosoma tekakahu	1	1	—	—
Oligosoma townsi	12	6	2.75 ± 0.42	0 – 5.0
Oligosoma waimatense	4	4	7.37 ± 0.89	0 – 11.0
Oligosoma whitakeri	7	3	2.3 ± 0.11	0 – 0.4
Oligosoma zelandicum	4	4	1.22 ± 0.33	0.6- 1.9
Oligosoma lichenigerum	2	1	0.14 ± 0.13	0.1

Reference

Hitchmough RA, Hoare JM, Jamieson H, Newman DG, Tocher MD, Anderson PJ, Lettink M, Whitaker AH, 2010. Conservation status of New Zealand reptiles, 2009. New Zealand Journal of Zoology 37:203-224.
