# Supplementary material for: A Retrospective Approach to Testing the DNA Barcoding Method
Source: PLoS One. 2013 Nov 11;8(11):e77882. doi: 10.1371/journal.pone.0077882 (PMC3823873; doi:10.1371/journal.pone.0077882)
Supplement: Figure S1 — Neighbour-joining tree (with 1000 bootstraps) for New Zealand skinks based on the 1977 taxonomy. Asterisks indicate the exemplar specimens for each species (See Table S2). The locality details are provided in Table S2. (PDF) [file pone.0077882.s007.pdf]

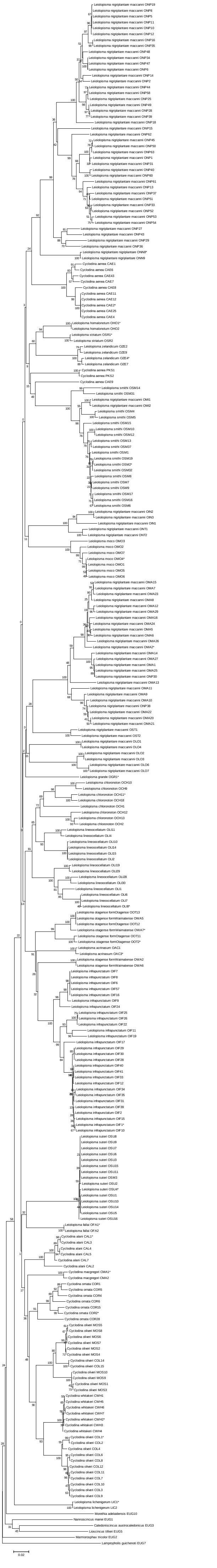
Leiolopisma nigriplantare maccanni ONP19
Leiolopisma nigriplantare maccanni ONP8
Leiolopisma nigriplantare maccanni ONP5
Leiolopisma nigriplantare maccanni ONP11
Leiolopisma nigriplantare maccanni ONP10
Leiolopisma nigriplantare maccanni ONP12
Leiolopisma nigriplantare maccanni ONP16
Leiolopisma nigriplantare maccanni ONP35
Leiolopisma nigriplantare maccanni ONP48
Leiolopisma nigriplantare maccanni ONP34
Leiolopisma nigriplantare maccanni ONP47
Leiolopisma nigriplantare maccanni ONP6
Leiolopisma nigriplantare maccanni ONP14
Leiolopisma nigriplantare maccanni ONP2
Leiolopisma nigriplantare maccanni ONP44
Leiolopisma nigriplantare maccanni ONP58
Leiolopisma nigriplantare maccanni ONP25
Leiolopisma nigriplantare maccanni ONP49
Leiolopisma nigriplantare maccanni ONP28
Leiolopisma nigriplantare maccanni ONP39
Leiolopisma nigriplantare maccanni ONP18
Leiolopisma nigriplantare maccanni ONP15
Leiolopisma nigriplantare maccanni ONP62
Leiolopisma nigriplantare maccanni ONP45
Leiolopisma nigriplantare maccanni ONP50
Leiolopisma nigriplantare maccanni ONP63
Leiolopisma nigriplantare maccanni ONP1
Leiolopisma nigriplantare maccanni ONP31
Leiolopisma nigriplantare maccanni ONP40
Leiolopisma nigriplantare maccanni ONP60
Leiolopisma nigriplantare maccanni ONP61
Leiolopisma nigriplantare maccanni ONP13
Leiolopisma nigriplantare maccanni ONP37
Leiolopisma nigriplantare maccanni ONP51
Leiolopisma nigriplantare maccanni ONP33
Leiolopisma nigriplantare maccanni ONP52
Leiolopisma nigriplantare maccanni ONP53
Leiolopisma nigriplantare maccanni ONP54
Leiolopisma nigriplantare maccanni ONP27
Leiolopisma nigriplantare maccanni ONP43
Leiolopisma nigriplantare maccanni ONP29
Leiolopisma nigriplantare maccanni ONP36
Leiolopisma nigriplantare nigriplantare ONN8*
Leiolopisma nigriplantare nigriplantare ONN9
Cyclodina aenea CAE1
Cyclodina aenea CAE6
Cyclodina aenea CAE43
Cyclodina aenea CAE7
Cyclodina aenea CAE8
Cyclodina aenea CAE11
Cyclodina aenea CAE12
Cyclodina aenea CAE2*
Cyclodina aenea CAE25
Cyclodina aenea CAE4
Leiolopisma homalonotum OHO1*
Leiolopisma homalonotum OHO2
Leiolopisma striatum OSR1*
Leiolopisma striatum OSR2
Leiolopisma zelandicum OZE2
Leiolopisma zelandicum OZE9
Leiolopisma zelandicum OZE4*
Leiolopisma zelandicum OZE7
Cyclodina aenea PKS1
Cyclodina aenea PKS2
Cyclodina aenea CAE9
Leiolopisma smithi OSM14
Leiolopisma smithi OSM31
Leiolopisma nigriplantare maccanni OMI1
Leiolopisma nigriplantare maccanni OMI2
Leiolopisma smithi OSM4
Leiolopisma smithi OSM5
Leiolopisma smithi OSM15
Leiolopisma smithi OSM10
Leiolopisma smithi OSM12
Leiolopisma smithi OSM13
Leiolopisma smithi OSM37
Leiolopisma smithi OSM1
Leiolopisma smithi OSM19
Leiolopisma smithi OSM2*
Leiolopisma smithi OSM32
Leiolopisma smithi OSM8
Leiolopisma smithi OSM7
Leiolopisma smithi OSM9
Leiolopisma smithi OSM17
Leiolopisma smithi OSM16
Leiolopisma smithi OSM6
Leiolopisma nigriplantare maccanni OIN2
Leiolopisma nigriplantare maccanni OIN3
Leiolopisma nigriplantare maccanni OIN1
Leiolopisma nigriplantare maccanni ONT1
Leiolopisma nigriplantare maccanni ONT2
Leiolopisma moco OMO3
Leiolopisma moco OMO2
Leiolopisma moco OMO7
Leiolopisma moco OMO4*
Leiolopisma moco OMO1
Leiolopisma moco OMO5
Leiolopisma moco OMO6
Leiolopisma nigriplantare maccanni OMA15
Leiolopisma nigriplantare maccanni OMA7
Leiolopisma nigriplantare maccanni OMA23
Leiolopisma nigriplantare maccanni OMA8
Leiolopisma nigriplantare maccanni OMA12
Leiolopisma nigriplantare maccanni OMA29
Leiolopisma nigriplantare maccanni OMA16
Leiolopisma nigriplantare maccanni OMA24
Leiolopisma nigriplantare maccanni OMA5
Leiolopisma nigriplantare maccanni OMA6
Leiolopisma nigriplantare maccanni OMA26
Leiolopisma nigriplantare maccanni OMA2*
Leiolopisma nigriplantare maccanni OMA14
Leiolopisma nigriplantare maccanni OMA27
Leiolopisma nigriplantare maccanni OMA1
Leiolopisma nigriplantare maccanni OMA25
Leiolopisma nigriplantare maccanni ONP30
Leiolopisma nigriplantare maccanni OMA13
Leiolopisma nigriplantare maccanni OMA11
Leiolopisma nigriplantare maccanni OMA9
Leiolopisma nigriplantare maccanni OMA10
Leiolopisma nigriplantare maccanni ONP38
Leiolopisma nigriplantare maccanni OMA22
Leiolopisma nigriplantare maccanni OMA20
Leiolopisma nigriplantare maccanni OMA21
Leiolopisma nigriplantare maccanni OST1
Leiolopisma nigriplantare maccanni OST2
Leiolopisma nigriplantare maccanni OLO1
Leiolopisma nigriplantare maccanni OLO4
Leiolopisma nigriplantare maccanni OLO2
Leiolopisma nigriplantare maccanni OLO3
Leiolopisma nigriplantare maccanni OLO6
Leiolopisma nigriplantare maccanni OLO7
Leiolopisma grande OGR1*
Leiolopisma chloronoton OCH10
Leiolopisma chloronoton OCH9
Leiolopisma chloronoton OCH11*
Leiolopisma chloronoton OCH18
Leiolopisma chloronoton OCH1
Leiolopisma chloronoton OCH12
Leiolopisma chloronoton OCH13
Leiolopisma chloronoton OCH2
Leiolopisma lineoocellatum OLI11
Leiolopisma lineoocellatum OLI4
Leiolopisma lineoocellatum OLI10
Leiolopisma lineoocellatum OLI14
Leiolopisma lineoocellatum OLI15
Leiolopisma lineoocellatum OLI2
Leiolopisma lineoocellatum OLI19
Leiolopisma lineoocellatum OLI29
Leiolopisma lineoocellatum OLI28
Leiolopisma lineoocellatum OLI30
Leiolopisma lineoocellatum OLI1
Leiolopisma lineoocellatum OLI6
Leiolopisma lineoocellatum OLI7
Leiolopisma lineoocellatum OLI8*
Leiolopisma otagense formOtagense OOT13
Leiolopisma otagense formWaimatense OWA5
Leiolopisma otagense formOtagense OOT12
Leiolopisma otagense formWaimatense OWA7*
Leiolopisma otagense formOtagense OOT11
Leiolopisma otagense formOtagense OOT2*
Leiolopisma acrinasum OAC1
Leiolopisma acrinasum OAC3*
Leiolopisma otagense formWaimatense OWA2
Leiolopisma otagense formWaimatense OWA6
Leiolopisma infrapunctatum OIF7
Leiolopisma infrapunctatum OIF8
Leiolopisma infrapunctatum OIF6
Leiolopisma infrapunctatum OIF57
Leiolopisma infrapunctatum OIF16
Leiolopisma infrapunctatum OIF9
Leiolopisma infrapunctatum OIF24
Leiolopisma infrapunctatum OIF25
Leiolopisma infrapunctatum OIF26
Leiolopisma infrapunctatum OIF22
Leiolopisma infrapunctatum OIF11
Leiolopisma infrapunctatum OIF19
Leiolopisma infrapunctatum OIF17
Leiolopisma infrapunctatum OIF29
Leiolopisma infrapunctatum OIF30
Leiolopisma infrapunctatum OIF28
Leiolopisma infrapunctatum OIF40
Leiolopisma infrapunctatum OIF41
Leiolopisma infrapunctatum OIF33
Leiolopisma infrapunctatum OIF12
Leiolopisma infrapunctatum OIF34
Leiolopisma infrapunctatum OIF35
Leiolopisma infrapunctatum OIF31
Leiolopisma infrapunctatum OIF39
Leiolopisma infrapunctatum OIF2
Leiolopisma infrapunctatum OIF15
Leiolopisma infrapunctatum OIF1*
Leiolopisma infrapunctatum OIF10
Leiolopisma suteri OSU8
Leiolopisma suteri OSU9
Leiolopisma suteri OSU7
Leiolopisma suteri OSU6
Leiolopisma suteri OSU3
Leiolopisma suteri OSU15
Leiolopisma suteri OSU11
Leiolopisma suteri OSM3
Leiolopisma suteri OSU2
Leiolopisma suteri OSU4*
Leiolopisma suteri OSU1
Leiolopisma suteri OSU10
Leiolopisma suteri OSU14
Leiolopisma suteri OSU5
Leiolopisma suteri OSU16
Leiolopisma fallai OFA1*
Leiolopisma fallai OFA2
Cyclodina alani CAL1*
Cyclodina alani CAL3
Cyclodina alani CAL4
Cyclodina alani CAL5
Cyclodina alani CAL7
Cyclodina alani CAL2
Cyclodina macgregori CMA1*
Cyclodina macgregori CMA2
Cyclodina ornata COR1
Cyclodina ornata COR5
Cyclodina ornata COR4
Cyclodina ornata COR6
Cyclodina ornata COR15
Cyclodina ornata COR2*
Cyclodina ornata COR28
Cyclodina oliveri MOS5
Cyclodina oliveri MOS8
Cyclodina oliveri MOS6
Cyclodina oliveri MOS7
Cyclodina oliveri MOS2
Cyclodina oliveri MOS4
Cyclodina oliveri COL14
Cyclodina oliveri COL15
Cyclodina oliveri MOS10
Cyclodina oliveri MOS9
Cyclodina oliveri MOS1
Cyclodina oliveri MOS3
Cyclodina whitakeri CWH1
Cyclodina whitakeri CWH5
Cyclodina whitakeri CWH6
Cyclodina whitakeri CWH7
Cyclodina whitakeri CWH2*
Cyclodina whitakeri CWH3
Cyclodina whitakeri CWH4
Cyclodina oliveri COL1*
Cyclodina oliveri COL2
Cyclodina oliveri COL4
Cyclodina oliveri COL6
Cyclodina oliveri COL8
Cyclodina oliveri COL12
Cyclodina oliveri COL11
Cyclodina oliveri COL7
Cyclodina oliveri COL10
Cyclodina oliveri COL3
Cyclodina oliveri COL9
Leiolopisma lichenigerum LIC1*
Leiolopisma lichenigerum LIC2
Morethia adelaidensis EUG10
Nannoscincus mariei EUG1
Caledoniscincus austrocaledonicus EUG3
Lioscincus tillieri EUG5
Marmorosphax tricolor EUG2
Lampropholis guichenoti EUG7
0.02

Fig. S1. Neighbour-Joining tree (with 1000 bootstraps) for New Zealand skinks based on the 1977 taxonomy. Asterisks indicate the exemplar specimens for each species (see Table S2). The locality details are provided in Table S2.
