# Supplementary material for: A Retrospective Approach to Testing the DNA Barcoding Method
Source: PLoS One. 2013 Nov 11;8(11):e77882. doi: 10.1371/journal.pone.0077882 (PMC3823873; doi:10.1371/journal.pone.0077882)
Supplement: Figure S2 — Neighbour-joining tree (with 1000 bootstraps) for New Zealand skinks based on the current taxonomy. Asterisks indicate the exemplar specimens for each species (See Table S2). The locality details are provided in Table S2. (PDF) [file pone.0077882.s008.pdf]

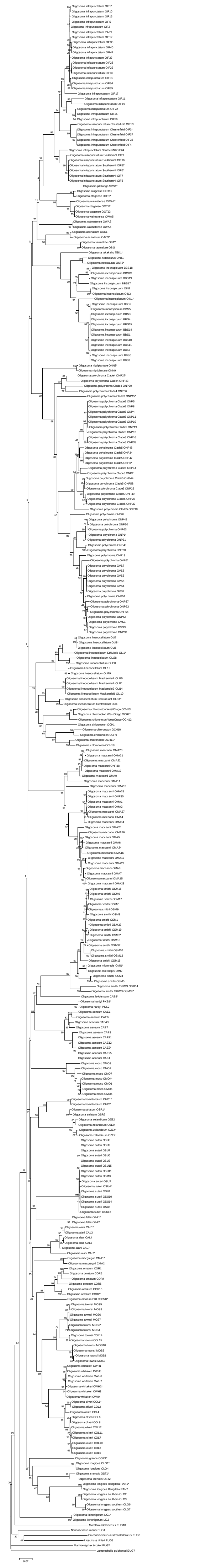

Fig. S2. Neighbour-Joining tree (with 1000 bootstraps) for New Zealand skinks based on the current taxonomy. Asterisks indicate the exemplar specimens for each species (see Table S2). The locality details for each sample are provided in Table S2.
